# Parallel genetic evolution and speciation from standing variation

**DOI:** 10.1002/evl3.106

**Published:** 2019-03-01

**Authors:** Ken A. Thompson, Matthew M. Osmond, Dolph Schluter

**Affiliations:** ^1^ Biodiversity Research Centre and Department of Zoology University of British Columbia Vancouver Canada; ^2^ Center for Population Biology University of California Davis California

**Keywords:** Adaptation, parallel evolution, speciation, theory

## Abstract

Adaptation often proceeds from standing variation, and natural selection acting on pairs of populations is a quantitative continuum ranging from parallel to divergent. Yet, it is unclear how the extent of parallel genetic evolution during adaptation from standing variation is affected by the difference in the direction of selection between populations. Nor is it clear whether the availability of standing variation for adaptation affects progress toward speciation in a manner that depends on the difference in the direction of selection. We conducted a theoretical study investigating these questions and have two primary findings. First, the extent of parallel genetic evolution between two populations rapidly declines as selection changes from fully parallel toward divergent, and this decline is steeper in organisms with more traits (i.e., greater dimensionality). This rapid decline happens because small differences in the direction of selection greatly reduce the fraction of alleles that are beneficial in both populations. For example, populations adapting to optima separated by an angle of 33° might have only 50% of potentially beneficial alleles in common. Second, relative to when adaptation is from only new mutation, adaptation from standing variation improves hybrid fitness under parallel selection and reduces hybrid fitness under divergent selection. Under parallel selection, genetic parallelism from standing variation reduces the phenotypic segregation variance in hybrids, thereby increasing mean fitness in the parental environment. Under divergent selection, larger pleiotropic effects of alleles fixed from standing variation cause maladaptive transgressive phenotypes when combined in hybrids. Adaptation from standing genetic variation therefore slows progress toward speciation under parallel selection and facilitates progress toward speciation under divergent selection.

**Impact summary**

Much of adaptation, especially that which occurs rapidly, proceeds from the sorting of ancestral standing variation and does not rely completely on *de novo* mutation. In addition, evolutionary biologists are increasingly embracing the fact that the difference in the direction of natural selection on pairs of populations is a quantitative continuum ranging from completely parallel to completely divergent. In this article, we ask two questions. First, how does the degree of genetic parallelism—here, adaptation using the same alleles in allopatric populations—depend on the differences in the direction of natural selection acting on two populations, from parallel (0°) to divergent (180°)? And second, how does adaptation from standing variation affect progress toward speciation, and does its effect depend on the direction of natural selection? We develop theory to address these questions. We first find that small differences in the direction of selection (angle) can largely preclude genetic parallelism. Second, we find that adaptation from standing variation has implications for speciation that change along the continuum from parallel to divergent selection. Under parallel selection, high genetic parallelism causes interpopulation hybrids to have high mean fitness when their parents adapt from standing variation. As selection tends toward divergent, adaptation from standing variation becomes less beneficial for hybrid fitness and under completely divergent selection causes interpopulation hybrids to have lower mean fitness than when adaptation was from new mutation alone. Our results provide general insight into patterns of genetic parallelism and speciation along the continuum of parallel to divergent natural selection.

In recent years, two general features of evolution by natural selection have become increasingly established. First, adaptation often proceeds largely via the reassortment of ancestral standing variation rather than via complete reliance on *de novo* mutations (Barrett and Schluter 2008). And second, variation in the direction of natural selection acting on pairs of populations is best represented by a quantitative continuum ranging from parallel selection—favoring identical phenotypes—to divergent selection—favoring distinct phenotypes—rather than falling into discrete “parallel” or “divergent” bins (Bolnick et al. 2018). It is unclear, however, how the extent of parallel genetic evolution—use of the same alleles during adaptation—changes with the difference in the direction of selection experienced by a pair of populations. It is also unclear whether adaptation from standing variation has implications for speciation that are distinct from those when adaptation is from new mutation alone, and whether its effect changes along the continuum from parallel to divergent natural selection. Here, we investigate genetic parallelism and speciation under adaptation from standing variation across this selection continuum.

Adaptation facilitates progress toward speciation when populations evolve reproductive isolating barriers as a by‐product. One reason these reproductive isolating barriers might arise is because genetic differences between populations have maladaptive consequences when combined in hybrids (i.e., postzygotic isolation), thereby reducing gene flow upon secondary contact. When a pair of populations adapts in response to divergent natural selection, hybrids might have an intermediate phenotype that is unfit in either parental environment (Schluter 2000). When a pair of populations is subject to parallel selection, they may diverge genetically by chance (Mani and Clarke 1990; Schluter 2009) and hybrids might have novel transgressive phenotypes that are poorly suited to the common parental habitat (Barton 1989). Hybrid unfitness is therefore determined by two factors: (1) additive gene action causing hybrids to “fall between the peaks” (Rundle and Whitlock 2001), and (2) cryptic genetic divergence that is released following hybridization and causes some hybrids to possess maladaptive transgressive phenotypes that vary in directions orthogonal to the axis of parental divergence (Arnegard et al. 2014; Keagy et al. 2016). How adaptation from standing variation affects progress toward speciation‐by‐selection (Langerhans and Riesch 2013) is largely unexplored theoretically.

Adaptation from standing variation is common and underlies some of the most spectacular adaptive radiations found in nature (Brawand et al. 2015). Genomic studies often implicate standing variation as the major source of genetic parallelism in replicate populations colonizing similar environments (Jones et al. 2012; Roesti et al. 2014; Lee and Coop 2017; Haenel et al. 2019) and adapting to novel stressors (Reid et al. 2016; Alves et al. 2019). Previous research has shown that the correlation between selection coefficients of a given allele in each of two populations inhabiting different environments is expected to increase with the similarity in the direction of selection (eq. 6 in Martin and Lenormand 2015). We therefore expected the extent of parallel genetic evolution for two populations to decline from a maximum to a minimum value as the angle between the directions of selection between them (θ) increases from completely parallel (*θ* = 0°) to completely divergent (*θ* = 180°). Our specific goal was to characterize the pattern of decline in parallelism. We also hypothesized that adaptation from standing variation would reduce the evolution of reproductive isolation under parallel selection because parental populations would fix more of the same alleles and therefore evolve fewer incompatibilities (Schluter 2009). Under divergent selection, we hypothesized that populations would fix alternative alleles regardless of whether they were selected from standing variation or new mutation. Therefore, we expected standing variation to have little effect on speciation by divergent selection compared to adaptation from new mutation alone.

We conducted a theoretical investigation into parallel genetic evolution and speciation from standing variation across the continuum from parallel to divergent natural selection. We primarily used individual‐based simulations and included some simple analytical arguments to gain intuition. We compared results from simulations where adaptation proceeds simultaneously via the sorting of ancestral standing genetic variation and *de novo* mutation to simulations where adaptation proceeds via *de novo* mutation alone. Our results provide insight into the circumstances under which we should expect high versus low genetic parallelism and also suggest that standing variation has substantial implications for speciation that depend on the difference in the direction of natural selection between populations.

## Methods

**Figure 1:**
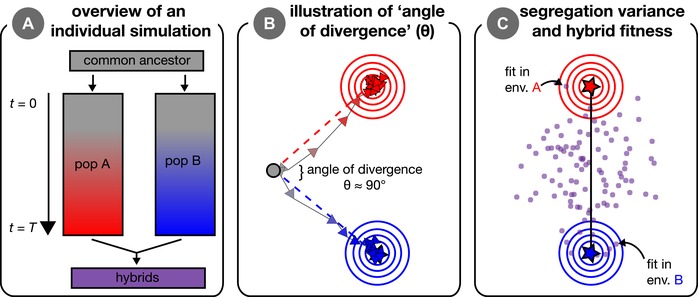
Visual overview of simulations and concepts. Panel (A) provides an overview of an individual simulation run. An ancestral population founds two initially identical parental populations that evolve independently for *T* generations in their respective environments. After *T* generations of adaptation, these parental populations interbreed to form hybrids. Panel (B) illustrates the process of adaptation in our simulations, wherein two populations (red and blue arrows connect the mean phenotype every 200 generations) independently adapt to specified optima (red and blue stars; behind arrows in [B] but visible in [C]). Concentric circles represent fitness contours around the two optima. The ancestor state is indicated by the gray dot, with the angle of divergence, *θ*, shown between the two axes of selection (red and blue dashed lines; angle shown is approximately 90°). Panel (C) illustrates the segregation variance in a group of hybrids. Individual hybrids (purple points) that are near an optimum have high fitness when measured in that environment. The black line is the line connecting parental optima—variance along this line can increase mean hybrid fitness whereas variance orthogonal to this line is deleterious.

We used computer simulations to investigate genetic parallelism and progress toward speciation—via ecologically dependent postzygotic reproductive isolation—from standing variation across the continuum from parallel to divergent natural selection. Our simulations consider pairs of populations and multivariate phenotypes determined by multiple additive loci. In each of our simulations, a single ancestral population founds two identical populations that each adapt in their respective environments without gene flow (i.e., allopatry; see Fig. 1A). After adaptation, populations interbreed to form recombinant hybrids. This general colonization history—a single population splitting into two populations that independently adapt to their respective novel environments—is modeled around the process of adaptation as it can occur in nature, for example in postglacial fishes (Bell and Foster 1994) and in birds or plants isolated within glacial refugia (e.g., Pettengill and Moeller 2012; Weir and Schluter 2004). In many such cases, ecologically dependent postzygotic isolation is thought to be essential for maintaining reproductive isolation (Nosil 2012). See Table 1 for descriptions of all parameters and values used in simulations.

**Table 1:** Description of parameters and parameter values in parental populations for simulations presented in the main text

Parameter	Value
*α*, mutation size SD in each dimension	0.1
*d*, distance between ancestral and parental phenotypic optima	1
*N*, number of haploid individuals	1000
*m*, number of traits, or ‘dimensionality’	5
*n*, initial number of segregating loci	0 (new mutation only) or 100 (new mutation and standing genetic variation)
*μ*, probability an individual acquires a new mutation	0.001
*σ*, strength of selection	1
*θ*, angle of divergence (°)	0 ≤ *θ* ≤ 180

### GENOTYPE TO PHENOTYPE

The phenotype of a haploid individual is represented by an *m*‐dimensional vector, **z** = [*z*_1_, *z*_2_,…, *z_m_*], with *m* being the number of uncorrelated “traits” or phenotypic “dimensions” (for further discussion of dimensionality, see Orr [2000] and Tenaillon [2014]). Each trait value, *z_i_*, is determined by the summed effects of alleles at all underlying loci (i.e., mutations act additively to determine the phenotype), which are initially fixed for alleles with an effect of 0 on all *m* traits. We primarily present results from simulations with five phenotypic dimensions (*m* = 5) in the main text. Results for alternative parameter combinations can be found in the supplementary figures (Figs. S1–S7).

### LIFE CYCLE

We model a Wright–Fisher population (Fisher 1930; Wright 1931) with haploid selection. Fitness is a Gaussian function that depends on the Euclidean distance between an individual's phenotype and the phenotypic optimum, ||**z** – **o**||, and the strength of selection, *σ* (e.g., Lande 1979):

(1) $$W=\exp(-\sigma {\left|\left|z-o\right|\right|}^{2}/2)$$

(Our qualitative conclusions are robust to alternative assumptions about fitness functions [see Fig. S8]). *N* haploid parents are then randomly sampled with replacement from a multinomial distribution with probabilities proportional to their fitness, *W*. Parents then randomly mate and produce two haploid offspring per pair, with free recombination between all loci. With probability *μ* an offspring gains a mutation; we assume an effectively infinite number of loci such that all mutations arise at a previously unmutated locus (“infinite‐sites” *sensu* Kimura [1969]). Mutational effects are drawn from a multivariate normal distribution (“continuum‐of‐alleles” *sensu* Kimura [1965]), with a mean of 0 and an SD of *α* in all *m* traits and no correlations among traits (i.e., universal pleiotropy).

### GENERATING STANDING GENETIC VARIATION

To generate ancestral standing variation, we conducted burn‐in simulations of a large ancestral population (*N*_anc_ = 10,000) under stabilizing selection (σ_anc_ = 0.01) at the origin (**o**_anc_ = [0, 0, … 0]) for 100,000 generations. All other parameters in the ancestor (e.g., mutation rate) were identical to those of parental populations (Table 1). This parameter combination facilitates the accumulation of appreciable standing variation (see Fig. S9), but our general conclusions hold if the ancestor is under much stronger selection (σ_anc_ = 1) that puts it into the multivariate “House‐of‐Cards” regime (Turelli 1985; see Fig. S10).

Ancestral populations reached mutation‐selection‐drift balance such that the rate of acquisition of new mutations was balanced by the rate of loss of mutations that arose in earlier generations (Fig. S9A). Both the mean frequency of derived alleles and the phenotypic (genotypic) variance were stable (Fig. S9B), as has been found in other models of phenotypes under stabilizing selection (e.g., Barton 1989). Segregating derived alleles were all at unique loci by assumption—that is, each polymorphic locus has exactly two alleles and each derived allele can be traced back to a single mutation event. In addition, segregating derived alleles were at low frequency in the ancestral population (see Fig. S9D for the site frequency spectrum). High derived allele frequencies and fixation are sometimes reached by drift when mutations have nearly neutral selective coefficients and by positive selection when mutations compensate for deleterious alleles that have risen to high frequency by drift (Hartl and Taubes 1996; Orr 2005).

### ADAPTATION TO A NEW ENVIRONMENT

In simulations with standing genetic variation, a parental population was established by first randomly choosing *n* polymorphic loci in the ancestor (see Fig. S11 for effect of *n* on genetic parallelism and segregation variance). Each parental individual received the mutant (i.e., “derived”) allele at each of these *n* loci with a probability equal to the allele's frequency in the ancestor. Loci fixed in the ancestral population were also fixed in the parental population but were not considered when quantifying parallelism. This admittedly artificial sampling procedure allowed us more control over the amount of standing genetic variation across simulations with different parameter values. Further control was achieved by making the second parental population initially identical to the first, so that each possessed the exact same collection of genotypes and there were therefore no founder effects. Populations adapted from only new (i.e., *de novo*) mutation when *n* = 0. Within each parameter combination, we began each replicate simulation from a unique realization of the ancestor (i.e., distinct burn‐in). After initialization, parental populations adapted to their respective phenotypic optima without interpopulation gene flow (Fig. 1B), and adaptation proceeded via natural selection on ancestral standing variation (if *n* > 0) and new mutation simultaneously.

Two properties of the new phenotypic optima are the key. The first is the Euclidean distance between each optimum and the origin, *d* (assumed the same for both parental populations for simulations presented in main text). More distant optima yield a greater amount of genetic and phenotypic change. In the main text, we set *d* = 1, which is equivalent to 10 times the SD of mutation effect size (*α*). The second key feature of the new optima is the angle of divergence, *θ*, between vectors that originate at the origin and each pass through one of the parental optima (dashed lines in Fig. 1B). Angle is used to quantify the difference in the direction of selection from parallel (*θ* = 0°) to divergent (*θ* = 180°) and is explicitly invoked in most empirical metrics that quantify phenotypic parallelism (see Bolnick et al. 2018). The value of *θ* determines the mean phenotypic differences that evolve between parental populations in our simulations (because *d* is held constant).

We ended the adaptation phase of simulations after *T* generations (*T* = 2000 in the main text), at which time all populations had reached their phenotypic optima (Fig. S12A) and mutation‐selection‐drift balance (Fig. S12B). An unavoidable and important effect of standing variation is that it quickens adaptation because populations do not have to wait for beneficial alleles to arise (Barrett and Schluter 2008). In our model and others like it (e.g., Barton 2001 and Chevin et al. 2014), reproductive isolation evolves rapidly during the initial stages of adaptation. After populations reach their respective phenotypic optima, genetic divergence accumulates slowly at a rate proportional to the mutation rate (Barton 1989, 2001; Chevin et al. 2014). Therefore, our results reflect quasi‐equilibrium conditions rather than transient states and are unaffected by standing variation's influence on the speed of adaptation.

### QUANTIFICATION OF GENETIC PARALLELISM AND HYBRID SEGREGATION VARIANCE AND FITNESS

To quantify parallel genetic evolution between parental populations, we first determined the number of alleles that fixed in each population (*f*_1_ and *f*_2_) and the number of alleles that fixed in both populations (*f*_1, 2_) during the adaptation phase. We then calculated our metric of “genetic parallelism” as:

(2) $$P_{g}=\frac{1}{2}\left(\frac{f_{1,2}}{f_{1}}+\frac{f_{1,2}}{f_{2}}\right)$$

Values of one indicate complete genetic parallelism (i.e., all alleles that fixed were fixed in both populations) and values of 0 indicate complete genetic nonparallelism (i.e., no allele fixed in both populations). We use this metric because of its ease of interpretation and note that it is highly correlated with other metrics of genetic divergence between populations (e.g., F_ST_; Fig. S13). We present some results with $P_{g}$ scaled between 0 and one, $\left[P_{g}-\min\left(P_{g}\right)\right]/\left[\max\left(P_{g}\right)-\min\left(P_{g}\right)\right]$, in order to facilitate comparison of simulations conducted with different parameters.

To create interpopulation hybrids, we then randomly sampled 100 individuals from each population without replacement. Each sampled individual was paired with an individual from the other population to form 100 unique interpopulation mating pairs. Every interpopulation mating pair then produced one recombinant haploid F_1_ hybrid for a total of 100 potentially unique hybrids.

After forming hybrids, we quantified their phenotypic variation—the net segregation variance (Wright 1968; Slatkin and Lande 1994)—calculated here as the mean phenotypic variance across all *m* traits. We present analyses of individual axes of variance where relevant. Higher segregation variance results when parents are differentiated by a greater number of alternative alleles (holding effect size constant) or alleles of individually larger effect (holding number of alleles constant) (Castle 1921; Slatkin and Lande 1994; Chevin et al. 2014). Segregation variance captures the phenotypic consequences of hybridization and has a direct impact on fitness whereas genetic (non)parallelism is only indirectly related to fitness. Phenotypic variance in parental populations (i.e., before hybridization) is near zero and does not differ between populations founded with versus without standing variation nor does it depend on the initial distance to the optimum (*d*; Fig. S12C). Such low variance is expected because our simulations have fixed optima, frequency‐independent selection, no migration, and parameter values corresponding to strong selection and relatively weak mutation (“house‐of‐cards”; Turelli 1984, 1985).

An individual hybrid's fitness in a given parental environment was calculated from its phenotype in the same manner as the fitness of parental populations (Fig. 1C). We determined the fitness (eq. (1)) of each hybrid in both parental environments and recorded its fitness as the larger of the two values. This can be imagined as, for example, giving the hybrid a choice of alternative host‐plants (see Drès and Mallet 2002) where it always chooses the host on which it has higher performance. Our fitness metric reflects what is traditionally recognized as “extrinsic” postzygotic isolation (Coyne and Orr 2004), and explicitly considers environment‐specific epistasis for fitness (Bateson 1909; Dobzhansky 1937; Muller 1942; Chevin et al. 2014; Fraïsse et al. 2016; see also Arnegard et al. 2014; Schumer et al. 2014; and Ono et al. 2017 for discussion of environment‐specific hybrid incompatibilities). We consider our model to be one of “extrinsic” rather than “intrinsic” isolation because we do not consider traits such as gamete viability, which experience environment‐independent selection. Rather, we imagine the traits in our model to be more akin to ecologically relevant traits like beak depth, under stabilizing selection with optima that depend on the environment. Because hybrids are recombinant, hybrid fitness reflects both the effects of displacement of the mean phenotype from the optimum (the “lag” load) and what in diploids is known as hybrid breakdown (Burton et al. 2006). We report hybrid fitness relative to an arbitrary parental population for each individual simulation, calculated as: [mean fitness of hybrids]/[mean fitness of parents].

## Results

### GENETIC PARALLELISM AND PHENOTYPIC SEGREGATION VARIANCE

**Figure 2:**
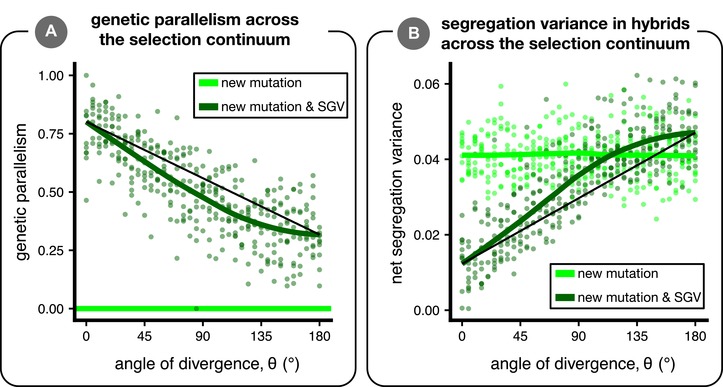
Genetic parallelism and phenotypic segregation variance. Parental populations adapted from either new mutation only (light green) or from a combination of new mutation and standing genetic variation (SGV) (dark green). Panel (A) shows $P_{g}$, the average fraction of fixed alleles that were also fixed in the other population (genetic parallelism; eq. (2)). The thin black line connects the fit at *θ* = 0° to the fit at *θ* = 180° and is shown only to facilitate visualization of the nonlinearity. Panel (B) is similar to (A), except with the net segregation variance in hybrids as the dependent variable. Plotted are the results from 10 replicate simulations for each of 37 angles of divergence (*d* = 1). Green lines are loess fits.

We first investigated how genetic parallelism between two populations—$P_{g}$, the average fraction of fixed alleles that were also fixed in the other population—changes with the angle of divergence (*θ*) when adaptation is from standing variation. Genetic parallelism is highest under completely parallel natural selection (*θ* = 0°) and rapidly decreases toward its minimum value as *θ* increases (dark green line and points in Fig. 2A; see black line for visual comparison of deviation from linearity). This rapid decrease in genetic parallelism also occurs when the phenotypic distance between parental optima is used as the independent variable instead of *θ*, although with our parameters nonlinearity is only appreciable in higher dimensions (see Fig. S14). There is considerable variation in genetic parallelism among simulation runs even when populations adapt to identical environments, which results from stochastic processes in each run. For example, alleles are lost due to drift, populations fix weakly deleterious alleles or different *de novo* mutations, and populations fix alternative alleles from the standing variation early in the simulations, which affects the selection coefficients of all other alleles in later generations (Chevin and Hospital 2008). Genetic parallelism rarely decreases to zero even under completely divergent selection (*θ* = 180°), indicating that populations fix some deleterious alleles. Our conclusion that genetic parallelism rapidly decreases with *θ* is generally robust to variation in population size and selection strength, except for when small populations are under weak selection (Fig. S1), likely due to an overwhelming effect of drift (see Fig. S15 for divergence between populations due to drift alone at various population sizes).

The changes in segregation variance generally mirror patterns of genetic parallelism (Fig. 2B). With standing variation, segregation variance is low under parallel selection and rapidly increases with *θ*. Chevin et al. (2014) found that segregation variance (proportional to their “variance load”) does not depend on *θ*, but in contrast to our model they did not permit genetic parallelism. When there is no standing variation, segregation variance is not affected by the angle of divergence (light green line and points in Fig. 2C; linear model slope ± 1 SE: −4.9 × 10^−7^ ± 5.8 × 10^−6^), in agreement with the findings of Chevin et al. (2014; their Fig. 2). At large angles, segregation variance is greater when populations adapt from standing variation than when they adapt from new mutation alone, and the magnitude of this difference increases with dimensionality (see Fig. S16).

**Figure 3:**
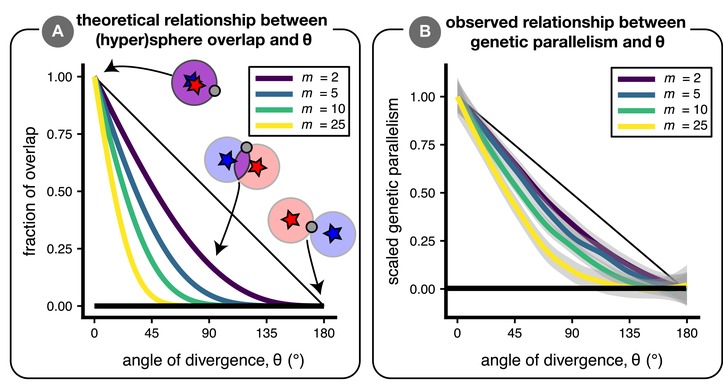
The relationship between trait dimensionality (*m*) and genetic parallelism. Panel (A) is an analytical result that depicts the relationship between *θ* and the fraction of overlap between two (hyper)spheres for four different dimensionalities (see eq. A1). In the inset cartoon, mutations that bring the phenotype into the red and blue regions are initially beneficial only in the “red” or “blue” environments, while mutations that bring the phenotype into the purple region are beneficial in both environments. The horizontal black line is set at 0 where there is no overlap. Panel (B) is a proof‐of‐concept figure showing loess fits of simulation results with 95% confidence intervals. Within a dimensionality, parallelism, $P_{g}$, is scaled between 0 (minimum value of loess fit) and 1 (maximum value of loess fit). Simulations were conducted with strong natural selection (*σ* = 10) to minimize the effect of drift. (See Fig. S19 for a similar result except with segregation variance on the *y*‐axis.)

Genetic parallelism decreases with *θ* (and segregation variance increases) because the fraction of alleles that are beneficial in both parental populations declines as *θ* increases. For a given population, beneficial alleles bring populations closer to the middle of a hypersphere centered at the phenotypic optimum (the geometric model of Fisher [1930]; see cartoon inset of Fig. 3A). Considering two populations, each with its own hypersphere, a given allele is beneficial in both—and thus could fix in parallel via positive natural selection—if it brings a population's mean phenotype into the region where the two hyperspheres overlap (purple region in Fig. 3A inset). The size of this region of overlap decreases rapidly with *θ* (Fig. 3A; see Appendix for mathematical details), and therefore so does the fraction of alleles present as standing variation that are beneficial in both populations. The rate of decrease of overlap is faster with greater dimensionality (Fig. 3A) but—perhaps surprisingly—does not depend on the distance to the optima (*d*; if *d*_1_ = *d*_2_ = *d*). Perhaps even more surprisingly, the fraction of overlap is not expected to change over the course of an “adaptive walk” (*sensu* Orr [1998]; see Appendix and Fig. A1 for detailed explanation). Briefly, this is because adaptation's effect is to shrink the radii of the hyperspheres (at roughly equivalent rates in the two populations if adaptation proceeds relatively deterministically). Thus, because the fraction of overlap (eq. A1) does not depend on the radii of the hyperspheres (*d*), the fraction of overlap is expected to remain constant throughout adaptation. Simulations conducted for four different dimensionalities (*m* = 2, 5, 10, 25) qualitatively capture the predicted pattern of decreasing parallelism with increasing dimensionality (Fig. 3B), although drift, a limited supply of standing variation, and run‐specific epistasis (etc.) contribute to quantitative differences between hypersphere overlap and genetic parallelism.

We also modeled an alternative case in which *θ* is held constant but populations differ in the distance to their respective optima (i.e., different vector “lengths” rather than “angles” *sensu* Bolnick et al. [2018]). Even if selection is completely parallel (i.e., *θ* = 0°), if the distance between the ancestral phenotype and the phenotypic optimum of population 2 is twice that of the ancestor‐optimum distance for population 1 (i.e., *d*_2_ = 2*d*_1_), less than 5% of the alleles beneficial to population 2 are also beneficial to population 1 (for *m* = 5; see Fig. S17). This result indicates that differences in vector lengths are important to consider—in addition to angles—for reducing the extent of genetic parallelism.

### HYBRID FITNESS

**Figure 4:**
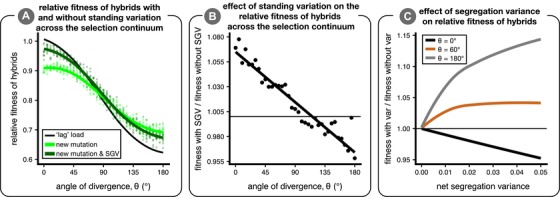
The effect of standing variation on mean hybrid fitness. Panel (A) shows the mean relative fitness of hybrids—as compared to parents—across environments in simulations initiated without (light green) and with (dark green) ancestral standing genetic variation. The thin black line represents the mean relative fitness of hybrids due only to the deviation of the observed mean phenotype from an optimum (“lag” load) and is close to 1 when the hybrid mean phenotype is on the optimum. Panel (B) shows the effect of standing variation on mean relative hybrid fitness (the ratio of values for dark/light green lines in panel [A]); the horizontal line shows where there is no effect of standing variation on relative mean hybrid fitness. Panel (C) is an analytical result that illustrates the relationship between segregation variance and mean hybrid fitness for three angles of divergence (black, *θ* = 0°; brown, *θ* = 60°; gray, *θ* = 180°) when the hybrid phenotype is multivariate normal with a mean exactly in between the two parental optima and equal variance in all phenotypic dimensions (no covariance). Hybrid fitness is plotted for each angle relative to the case of no variance; the horizontal line indicates when segregation variance has no effect on hybrid fitness.

In this section, we evaluate the effect of standing variation on hybrid fitness across the continuum from parallel to divergent natural selection. The most readily observable pattern is that the mean relative fitness of hybrids is lower under divergent selection than under parallel selection regardless of whether adaptation proceeds with standing variation (Fig. 4A). This pattern occurs because the hybrid mean phenotype is increasingly distant from either parental optimum as *θ* increases. In Figure 4A, we plot the fitness of the hybrid mean phenotype (representing the “lag” load) as a thin black line.

**Figure 5:**
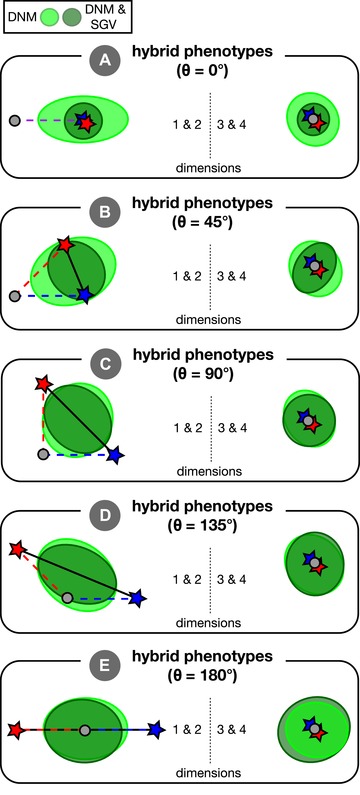
The effect of standing variation on the distribution of hybrid phenotypes. We plot ellipses containing 95% of hybrid phenotypes for five angles of divergence (*θ*) evenly spaced along the continuum of (A) completely parallel (*θ* = 0°) to (E) completely divergent (*θ* = 180°) selection. Separate ellipses are shown for simulations where populations adapted from only new mutation (light green; DNM) or both new mutation and standing genetic variation (dark green; DNM and SGV). Each ellipse is fit to 1000 hybrids resulting from 10 replicate simulations. Parental optima are depicted as stars and the origin (ancestral optimum) is shown as a grey dot. The left side of each panel shows the first two trait dimensions—the only dimensions in which the optima might differ. The right side of each panel shows the third and fourth dimensions—both of which are under stabilizing selection for a phenotype identical to the ancestral optimum. The axes of selection connect the origin and optima (dashed red and blue lines) and we also show the axis connecting parental optima as a solid black line. Ellipse plotting order is reversed on the right side of panel (E) to facilitate visualization.

Compared to when adaptation is from new mutation, adaptation from standing variation improves mean hybrid fitness when parental populations adapt to similar optima but reduces hybrid fitness when parents undergo divergent adaptation (Fig. 4B). This pattern is caused by environment‐specific effects of segregation variance on mean hybrid fitness (Fig. 4C and Fig. 5). When the hybrid phenotype distribution is centered at the phenotypic optimum—as it is under parallel selection (*θ* = 0°)—segregation variance is universally deleterious. When parental populations adapt to identical optima from only new mutation, hybrids vary considerably around the parental optimum and thus have relatively low mean fitness. When populations have access to a common pool of standing variation, parallel genetic evolution leads to lower segregation variance around the optimum and therefore higher mean fitness under parallel selection compared to when populations adapt from only new mutation (Fig. 5A; see Fig. S18 for similar results but for maximum hybrid fitness instead of mean).

At large angles of divergence, adaptation from standing variation reduces hybrid fitness compared to when adaptation is from only new mutation. The reasons for this are twofold. First, since we allow hybrids to “choose” their environment (measuring their fitness in the parental environment they are better adapted to), at larger angles hybrids increasingly fall into a “fitness valley.” In this case, some variation along the axis of divergence can be beneficial (see Fig. 4C). Second, since fitness in either environment is a Gaussian function, variation becomes beneficial when the mean is far from the optimum (by Jensen's inequality), even when considering fitness in only a single environment. This result is robust to variation in parameter values (see Figs. S4–S6), except when selection is very weak in small populations.

There are appreciable differences in patterns of phenotypic variation in hybrids when their parents adapt with standing variation versus when adaptation is from new mutation alone (Fig. 5). Only phenotypic variation along the axis connecting parental optima (black line connecting stars in Fig. 5) can be beneficial, whereas variation along orthogonal axes is always deleterious. When *θ* = 180°, standing genetic variation reduces hybrid variation along the axis connecting parental optima but slightly increases variation along all other axes (see Fig. 5E). Thus, adaptation from standing variation increases maladaptive segregation variance—due to cryptic genetic differences between parental populations revealed only after hybridization—and thereby reduces hybrid fitness under large angles of divergence.

**Figure 6:**
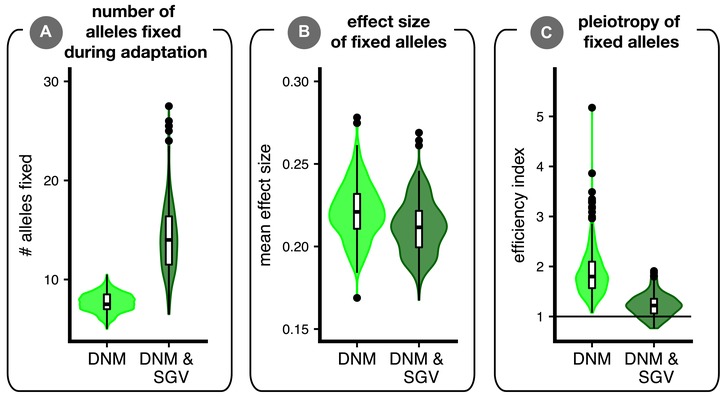
Properties of alleles fixed during adaptation. We show results from simulations where parental populations adapted from only *de novo* mutation (light green; DNM) versus adaptation from standing variation and new mutation (dark green; DNM & SGV). Each replicate simulation contributed one data point to the plot. Panel (A) shows the average number of alleles fixed during adaptation. Panel (B) shows the average effect size (Euclidean length of mutation vector) of alleles fixed during adaptation. Panel (C) shows the allele “efficiency index” for a parental population, the ratio of fixed mutations’ mean effect sizes along the axis of selection versus their mean effect size averaged across all orthogonal directions. Values of 1 (horizontal line) are equally balanced in these directions, and mutations are more “efficient” (i.e., they point more directly at the optimum) as this index increases. Statistical tests confirm all differences as highly significant (not shown).

Why does adaptation from standing variation alter patterns of phenotypic segregation variance in hybrids? As discussed above, adaptation from standing genetic variation reduces segregation variance under parallel selection because parents fix the same alleles that therefore do not segregate in hybrids. Populations adapting from standing variation also fix a greater number (Fig. 6A) of smaller effect alleles (Fig. 6B) than populations evolving without standing variation. Fixation of smaller effect alleles likely occurs under adaptation from standing variation because stabilizing selection in the ancestor effectively removes large‐effect alleles from the standing variation (Fig. S9) and because weakly beneficial alleles have a higher probability of fixation when present in standing variation compared to if they arose *de novo* (Orr and Betancourt 2001; Hermisson and Pennings 2005; Matuszewski et al. 2015).

This latter effect seemed to allow alleles with more deleterious pleiotropic effects to fix during adaptation from standing variation than when adaptation was from new mutation alone (Fig. 6C). That is, populations initiated with standing genetic variation used alleles with proportionally larger pleiotropic side effects. We quantified pleiotropy in a parental population by taking the ratio of the mean effect size of fixed alleles along the axis of selection (red or blue dashed lines in Fig. 5) versus the mean effect size of fixed alleles averaged across all orthogonal axes, termed the “efficiency index.” Values of 1 (horizontal line in Fig. 6C) imply that, on average, alleles had equivalent effects along the axis of selection as they did along each orthogonal axis. Increasingly positive values reflect the presence of alleles that take a population to the optimum more “efficiently” (i.e., directly along the dashed blue or red line in Fig. 5). Together, these results indicate that adaptive walks from standing variation in our simulations involved more—slightly smaller—steps and are more “meandering” than adaptive walks from new mutation alone, which use fewer—slightly larger—and more direct steps (but see Ralph and Coop 2015). These differences in the properties of alleles fixed in simulations initiated with versus without standing variation contribute to the patterns of phenotypic segregation variance that ultimately determine the fitness of hybrids.

## Discussion

In this study, we investigated parallel genetic evolution and progress toward speciation under adaptation from standing variation. We characterized how the extent of genetic parallelism from standing variation changes with the angle of divergence between parental optima, then illustrated how adaptation from standing variation affects hybrid fitness under various forms of natural selection. Here, we highlight our key findings, predictions for empirical systems, and suggestions for future work.

### KEY PREDICTIONS AND POSSIBLE TESTS

The first principal finding of our study is that the degree of genetic parallelism rapidly declines as the angle of divergence increases from parallel toward divergent, especially when a large number of traits affect fitness. Practically, this means that the extent of genetic parallelism should decline quickly with phenotypic divergence. It is possible to test this prediction in natural or experimental populations using techniques such as “Phenotypic Change Vector Analysis,” which estimate important parameters such as the angle between the vectors and/or the difference in their magnitudes (Bolnick et al. 2018). (Of course, phenotypic measurements are imperfect and typically noncomprehensive, and accordingly estimates of interpopulation divergence are necessarily made with some error.) Natural systems exhibiting repeated instances of easily‐quantified phenotypic divergence (see Oke et al. 2017; Stuart et al. 2017) are amenable to this approach. Given that phenotypic and genetic parallelism are unlikely to be linearly related (Fig. S14), we suggest careful consideration when generating predictions for empirical systems. We also note that studies quantifying genetic parallelism (e.g., Jones et al. [2012]) typically do not quantify nonparallel changes. To test our predictions about genetic parallelism (Fig. 3), it will be necessary for future studies to measure both the number of parallel genetic changes (*f*_1, 2_ in eq. (2)) and the total number of genetic changes (*f*_1_ and/or *f*_2_ in eq. (2)) in pairs of populations being compared (as in Alves et al. [2019]).

Our second principal finding is that—relative to when adaptation is from only *de novo* mutation—adaptation from standing genetic variation improves the mean fitness of hybrids under parallel natural selection, has little effect at intermediate angles of divergence, and reduces mean hybrid fitness under completely divergent selection. Practically, this indicates that adaptation from standing variation works against “mutation‐order” speciation and facilitates “ecological” speciation (Schluter 2009; Schluter and Conte 2009). This hypothesis could be tested most readily in experimental systems where the amount of ancestral standing variation can be easily manipulated, and where interpopulation hybrids can easily be generated to have their fitness measured in parental environments. It would also be worthwhile to empirically test whether alleles fixed from standing variation are indeed more pleiotropic than alleles fixed from *de novo* mutation, as predicted by our simulations.

We emphasize that the mechanism through which adaptation from standing variation affects hybrid fitness (relative to adaptation from *de novo* mutation) differs between simulations where populations adapted under parallel versus divergent selection. Under parallel selection, standing variation's effect on hybrid fitness is caused largely by parallel genetic evolution and therefore adaptation from standing variation is most likely to have an effect if populations adapting in parallel are founded with the same standing variation. Under divergent selection, standing variation's effect on hybrid fitness is not caused by genetic parallelism but rather by cryptic genetic differences—cryptic because they don't reveal themselves until after hybridization—that evolve between parental populations. Therefore, our predictions about the effect of adaptation from standing variation on hybrid fitness under divergent selection should hold regardless of whether populations have the same or different initial standing variation. A simple prediction—testable theoretically and empirically—resulting from our study is that founder effects should have a greater effect on hybrid fitness under parallel selection than under divergent selection.

### ALTERNATIVE SOURCES OF STANDING VARIATION

Our model addresses the case of adaptation from a pool of standing genetic variation at mutation‐selection‐drift balance. This framework does not address cases of adaptation where standing variation is generated from other sources. For example, in threespine stickleback, the marine ancestral form maintains standing variation for freshwater‐adapted alleles in a balance between migration of alleles from freshwater populations and negative selection in the sea (the “transporter” hypothesis; Schluter and Conte 2009; Nelson and Cresko 2018). In this case, the pool of standing variation is enriched for alleles that have already swept to high frequencies in freshwater populations—that is, they are “pretested” by selection. Scenarios such as this are especially likely to lead to genetic parallelism (Schluter and Conte 2009). The extent to which adaptation from standing variation proceeds via the sorting of naïve alleles (as in our model) versus pretested alleles (as in the transporter model) is unresolved.

### POSSIBLE EXTENSIONS

Some of our conclusions will change under alternative assumptions. Some assumptions—for example a lack of recurrent *de novo* mutation or gene flow—reduce the extent of genetic parallelism (Nosil and Flaxman 2011; Anderson and Harmon 2014; Ralph and Coop 2015; Barghi et al. 2019). We also assumed universal pleiotropy and future work examining the effect of modularity on our results—especially on changes in parallelism with the angle of divergence—would be valuable. In addition, we considered only haploid selection, had only additive effects of alleles on phenotypes, and assumed that the sole fitness optima available to hybrids are those to which the two parents are adapted. Our analytical results also ignore variation in the probability that particular mutations arise and fix (or are present as standing variation). Extending our analytical approach to integrate the distribution of fitness effects of new mutations (Eyre‐Walker and Keightley 2007), the correlation of selection coefficients across environments (Kassen 2014; Martin and Lenormand 2015), and existing theory on the probability of genetic parallelism from standing variation (MacPherson and Nuismer 2017) will be valuable.

We also note that the only reproductive isolating barrier we considered was environment‐specific postzygotic isolation. Postzygotic isolation can also be environment‐independent, and such “intrinsic” isolating barriers are correlated with genetic divergence between populations (Orr 1995; Matute et al. 2010; Moyle and Nakazato 2010; Wang et al. 2015). Therefore, our measure of genetic parallelism might be interpreted as being inversely proportional to the strength of intrinsic barriers. We also did not consider prezygotic barriers such as assortative mating (Gavrilets 2004). Accordingly, our results might be most relevant for empirical systems where ecology‐based postzygotic isolation has a primary role in the origin of species.

### CONCLUDING REMARKS

In this study, we characterized patterns of genetic parallelism and progress toward speciation from standing variation in pairs of populations with quantitative differences in the direction of selection between them. Our findings generate new hypotheses for empirical studies on genetic parallelism and speciation. As evolutionary biologists develop increasingly powerful tools for detecting parallel genetic adaptation in nature, it will be important to keep in mind that genetic parallelism could be less common than we might intuit from patterns of selection and phenotypic similarity. We have also shown that adaptation from standing variation is expected to weaken the strength of isolating barriers that evolve between populations subject to parallel natural selection. By contrast, adaptation from standing variation can facilitate the process of speciation via divergent natural selection (i.e., “ecological” speciation), suggesting that adaptation from standing variation might have a role in adaptive radiation beyond simply increasing the rate at which adaptation proceeds.

Associate Editor: Z. Gompert

## Supporting information


**Appendix**
**Fig. A1**. Cartoon illustration of why divergence among populations does not affect whether an allele is beneficial in both of them.
**Figure S1**. Genetic parallelism across the continuum of parallel to divergent natural selection (*N* = 100).
**Figure S2**. Genetic parallelism across the continuum of parallel to divergent natural selection (*N* = 1000).
**Figure S3**. Genetic parallelism across the continuum of parallel to divergent natural selection (*N* = 5000).
**Figure S4**. Effect of standing genetic variation on hybrid fitness across the continuum of parallel to divergent natural selection (*N* = 100).
**Figure S5**. Effect of standing genetic variation on hybrid fitness across the continuum of parallel to divergent natural selection (*N* = 1000).
**Figure S6**. Effect of standing genetic variation on hybrid fitness across the continuum of parallel to divergent natural selection (*N* = 5000).
**Figure S7**. Properties of fixed mutations under a variety of parameter combinations (*N* = 1000).
**Figure S8**. Simulations under various fitness functions.
**Figure S9**. Mutation‐selection balance and mutation effect sizes in ancestral populations.
**Figure S10**. Mutation‐selection balance and mutation effect sizes in ancestral populations under stronger selection (*σ*_anc_ = 1).
**Figure S11**. The effects of standing genetic variation on genetic parallelism and phenotypic segregation variance in hybrids under parallel and divergent natural selection.
**Figure S12**. Effect of standing variation on the pace of adaptation and attainment of mutation‐selection‐drift balance.
**Figure S13**. Relationship between genetic parallelism and (A) segregation variance and (B) expected heterozygosity.
**Figure S14**. Alternative presentation of simulation results across environments: distance between optima (*δ*).
**Figure S15**. The effect of population size on the rate of divergence between populations due to drift.
**Figure S16**. Effect of dimensionality on net segregation variance.
**Figure S17**. Fraction of overlap of beneficial mutations with parallel selection (*θ* = 0°) but unequal distance (*d*_1_ ≠ *d*_2_).
**Figure S18**. The effect of standing genetic variation (SGV) on relative maximum hybrid fitness across environments.
**Figure S19**. The relationship between segregation variance and *θ* for different dimensionalities.

## Data Availability

Python (version 3.6.4) simulation scripts and resulting data, R (version 3.4.1) scripts to process and plot the simulated data, and Mathematica notebooks (version 9) to derive analytical results are available on Dryad (https://doi.org/10.5061/dryad.g68d124).
