# Supplementary material for: Parallel genetic evolution and speciation from standing variation
Source: Evol Lett. 2019 Mar 1;3(2):129–41. doi: 10.1002/evl3.106 (PMC6591551; doi:10.1002/evl3.106)
Supplement: Supplementary file 1 — Appendix Fig. A1. Cartoon illustration of why divergence among populations does not affect whether an allele is beneficial in both of them. Figure S1. Genetic parallelism across the continuum of parallel to divergent natural selection (N = 100). Figure S2. Genetic parallelism across the continuum of parallel to divergent natural selection (N = 1000). Figure S3. Genetic parallelism across the continuum of parallel to divergent natural selection (N = 5000). Figure S4. Effect of standing genetic variation on hybrid fitness across the continuum of parallel to divergent natural selection (N = 100). Figure S5. Effect of standing genetic variation on hybrid fitness across the continuum of parallel to divergent natural selection (N = 1000). Figure S6. Effect of standing genetic variation on hybrid fitness across the continuum of parallel to divergent natural selection (N = 5000). Figure S7. Properties of fixed mutations under a variety of parameter combinations (N = 1000). Figure S8. Simulations under various fitness functions. Figure S9. Mutation‐selection balance and mutation effect sizes in ancestral populations. Figure S10. Mutation‐selection balance and mutation effect sizes in ancestral populations under stronger selection (σ anc = 1). Figure S11. The effects of standing genetic variation on genetic parallelism and phenotypic segregation variance in hybrids under parallel and divergent natural selection. Figure S12. Effect of standing variation on the pace of adaptation and attainment of mutation‐selection‐drift balance. Figure S13. Relationship between genetic parallelism and (A) segregation variance and (B) expected heterozygosity. Figure S14. Alternative presentation of simulation results across environments: distance between optima (δ). Figure S15. The effect of population size on the rate of divergence between populations due to drift. Figure S16. Effect of dimensionality on net segregation variance. Figure S17. Fraction of overlap of beneficial mutations with pa [file EVL3-3-129-s001.pdf]

Supporting information for:

Patterns of speciation and parallel genetic adaptation from standing variation

by

Ken A. Thompson, Matthew M. Osmond and Dolph Schluter

Contents:

Appendix

Fig. A1

Figures S1-18

Appendix

Here we outline an explanation for why genetic parallelism decreases rapidly with the angle of divergence, θ (Fig. 2A, and distance between optima (Fig. S15B). Our explanation focuses on the extent of phenotypic space wherein mutations improve the fitness of both adapting populations in their respective environments. At the time of founding both adapting populations have the same mean phenotype, which is the mean ancestral phenotype. Mutations that move this ancestral mean phenotype into the region that leads to higher fitness in both parental environments are thus beneficial in both populations. The region of phenotypic space that has higher fitness than the mean phenotype in one environment is a hypersphere (of dimension m), centred on the optimum with a radius equal to the distance between the mean phenotype and the optimum, d. A similar hypersphere characterizes the phenotypic space that has higher fitness than the mean phenotype in the other parental environment. The region that is mutually beneficial is then the intersection of two hyperspheres, which is the union of two hyperspherical caps.

Fortunately, the volume of a hyperspherical cap is known for any dimension, m (Li 2011). It depends on the dimensionality (m), the radii of the two hyperspheres (D), and the distance between their centers (δ). In our case the distance between the two centres is $\delta = 2d * \sin(\theta/2)$. The amount of phenotypic space that is beneficial in a given environment is simply the volume of one of the hyperspheres. Thus, dividing the volume of the mutually-beneficial space (the union of the hyperspherical caps) by the volume of the space beneficial in a given environment (one of the hyperspheres) gives the fraction of beneficial mutations which are mutually beneficial. Using the formula given by Li (2011; their eqn 3) for the volume of a hyperspherical cap created by the intersection of two m-dimensional hyperspheres with radii d

whose centres are distance $\delta = 2d * \sin(\theta/2)$ apart, the fraction of beneficial mutations that are expected to be beneficial in both is:

$$I_x[(1 + m)/2, 1/2] \quad \text{(A1)}$$

where $I_x[a, b]$ is the regularized incomplete beta function (Equation 6.6.2 in Abramowitz and Stegun [1972]) and here $x = \text{Cos}(\theta/2)^2$. Eq. A1 depends on only m and θ, that is the solution is independent of the distance from the ancestor to the new optima, d. We refer to Eq. A1 as the fraction of overlap in the main text, but note that this is only true when $d_1 = d_2$ (the formula is more complex when $d_1 \neq d_2$, but can easily be used, e.g., Fig. S16B). The incomplete regularized beta function arises from integrating $\sin^m(\theta)$ over θ (Li 2011).

The solution of Eq. A1 exhibits a rapid decrease with θ for all values of $m > 0$, and the decrease is faster for greater values of m (Fig. 2B). Thus, if standing genetic variation was uniformly distributed throughout the beneficial hyperspheres, the percent of segregating beneficial mutations that were beneficial in both parental populations, and thus expected to potentially fix in both, would decrease rapidly with the angle of divergence.

The above analysis considers only the very onset of adaptation, when the two parental populations have the same mean phenotypes, such that the fraction of phenotypic space that is beneficial in one population that is also beneficial in the other population (call this X) is equivalent to the fraction of possible beneficial mutations (if uniformly distributed across the hyperspheres) that are beneficial in both populations (call this Y). As adaptation proceeds the mean phenotypes of the parental populations depart from one another and X therefore no longer equals Y. This is because mutations are vectors that move a phenotype in a particular direction, and thus a mutually beneficial point in phenotypic space is only guaranteed to be a mutually beneficial mutation if both populations have the same mean phenotype.

To account for the inequality between phenotypic space (*X*) and mutational vectors (*Y*) during adaptation we must shift the mean phenotypes so that they are at the same point in phenotypic space and move their optima by an identical translation (see Fig. A1). We then have $X=Y$. One way to imagine this is to keep the mean phenotypes in place at the mean ancestral phenotype (the origin) and consider adaptation as the movement of the optima closer to the mean phenotypes. From this perspective, adaptation's effect is a shrinking of the radii of the hyperspheres (at roughly equivalent rates in the two populations if adaptation proceeds relatively deterministically). Thus, because the fraction of overlap (Eq. A1) does not depend on the radii of the hyperspheres, the fraction of overlap is expected to remain constant throughout adaptation.

In reality and in our simulations, standing genetic variation is not uniformly distributed, the probability of fixation varies across the region of overlap, and adaptation uses up some of the standing variation so that the distribution of standing variation changes with time. Taking the first two complications into account would require weighted averages across the space contained in the hyperspherical caps, which is beyond the scope of our study. The third complication is yet more involved and would require an analysis of how standing genetic variation is used as adaptation proceeds (i.e., how the distribution of segregating effects and allele frequencies shift as alleles fix). Such a calculation is also beyond the scope of this article. Despite these complications, it seems as though the simple analysis above qualitatively captures the essence of why genetic parallelism decreases rapidly with the angle of divergence.

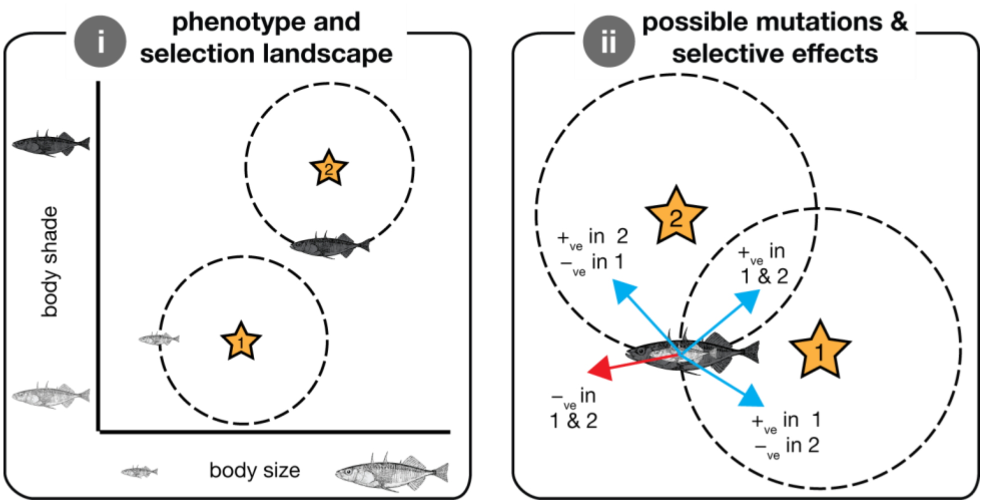

Fig. A1. Cartoon illustration of why divergence among populations does not affect whether an allele is beneficial in both of them. Panel (i) depicts the phenotype landscape and selection landscape. Variation in the horizontal dimension reflects phenotypic variation in body size, and the vertical dimension reflects variation in body shade. We depict two 'populations' with differences in body size and shade (small & light; big & dark). The stars reflect local optima after a hypothetical environmental shift—selection favours adaptation toward a larger body size in population 1 and selection for darker body shade in population 2. If we illustrate the circle of beneficial mutational space (dashed circles) with respect to the current phenotypic position they do not overlap. Panel (ii) illustrates the selection landscape as it is 'experienced' by each population. An allele that slightly increases body size and darkens the body shade from the current phenotype (the position of the fish cartoons) is beneficial (blue) in both of populations. Some alleles are beneficial in only one population, and others are deleterious in both (red). Thus, even though the spheres do not overlap in (i) it is not the case that they populations will undergo non-parallel genetic evolution.

Supplementary figures

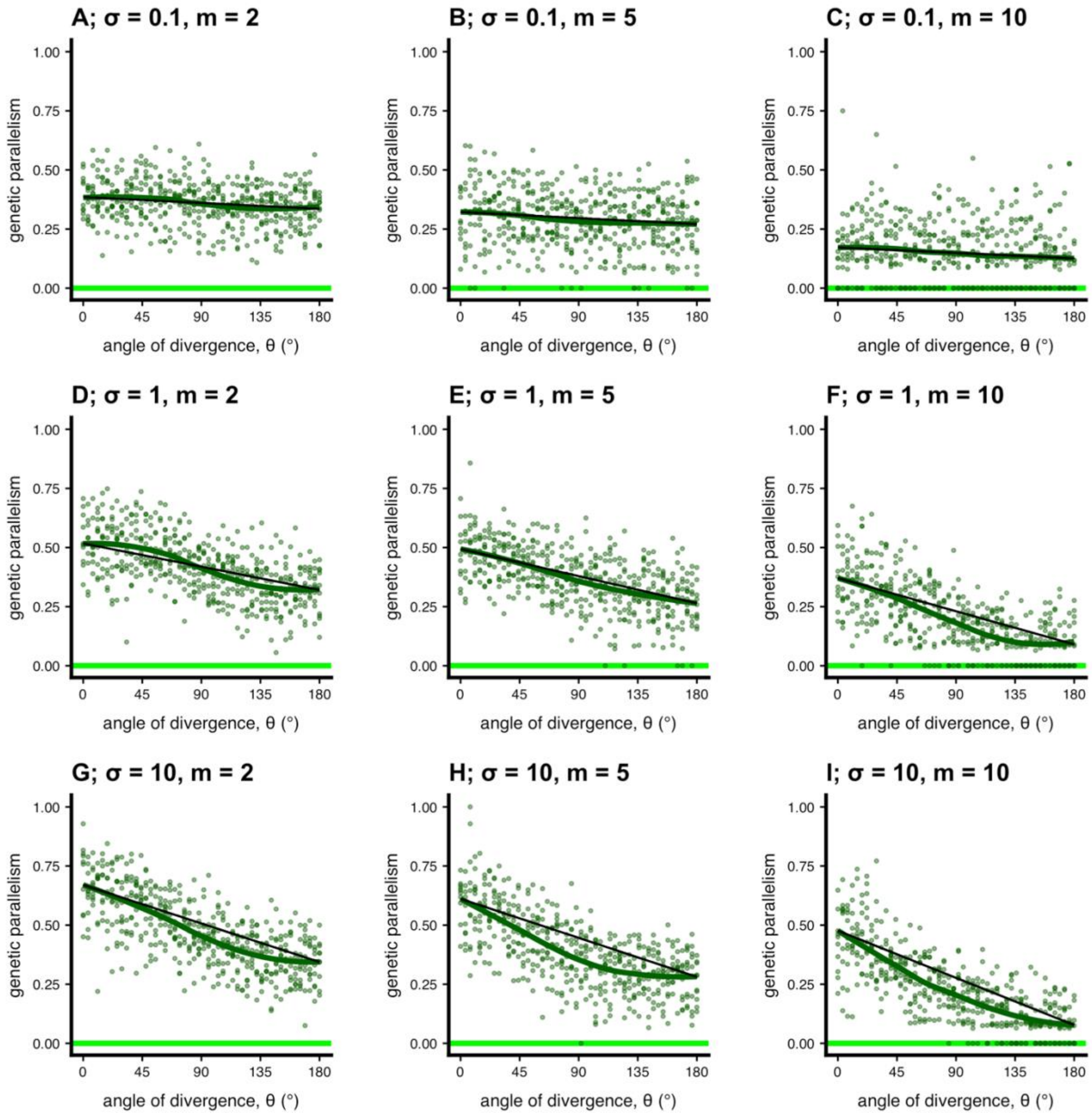

Figure S1. Genetic parallelism across the continuum of parallel to divergent natural selection (N = 100). This figure presents simulations similar to Fig. 2A in the main text but with varying parameter values (selection [σ] and dimensionality [m]). We ran these particular simulations for T = 5000 generations. All other parameters as in main text.

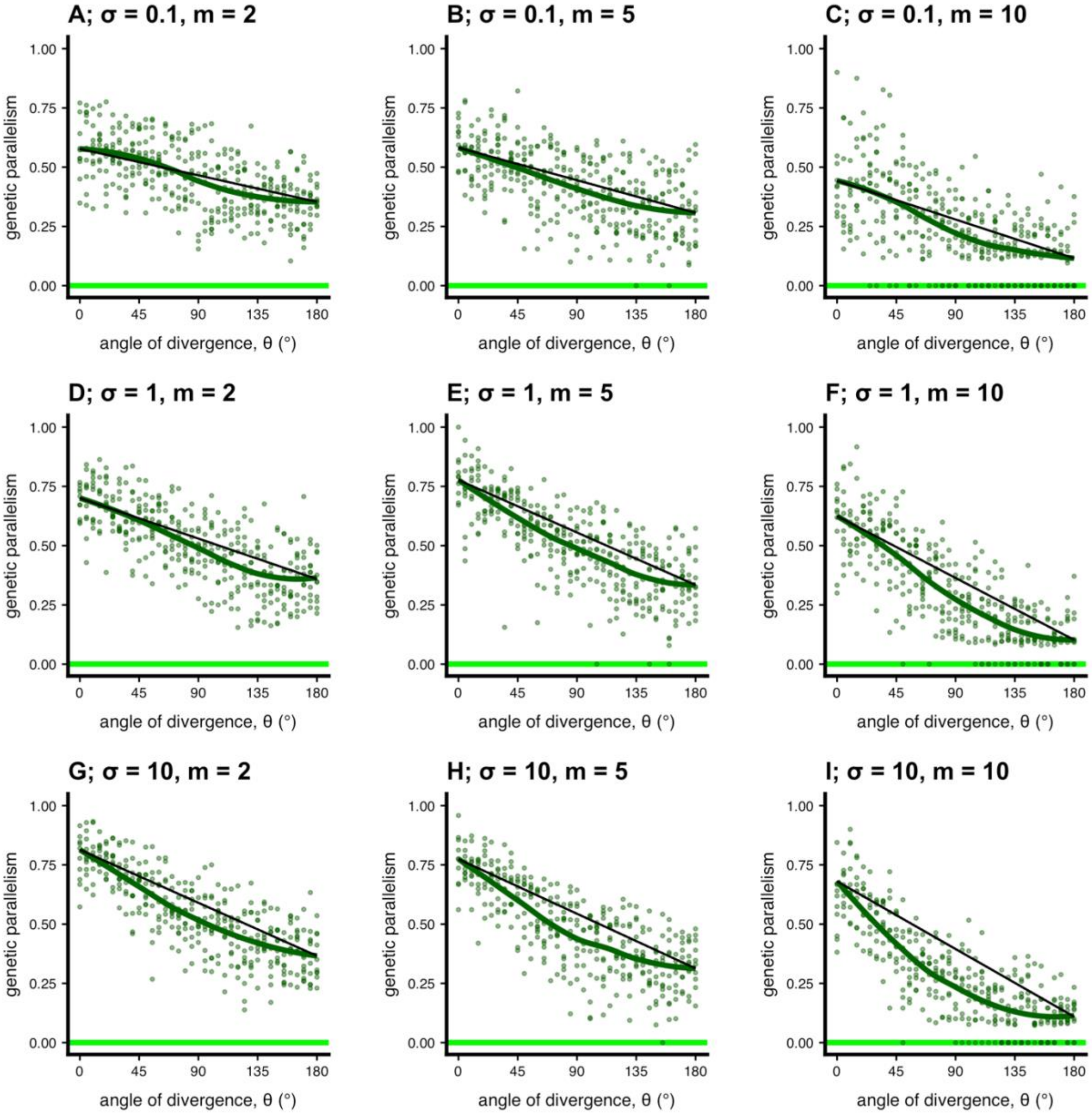

Figure S2. Genetic parallelism across the continuum of parallel to divergent natural selection (N = 1000). This figure presents simulations similar to Fig. 2A in the main text but with varying parameter values (selection [σ] and dimensionality [m]). We ran these particular simulations for T = 2000 generations. All other parameters as in main text.

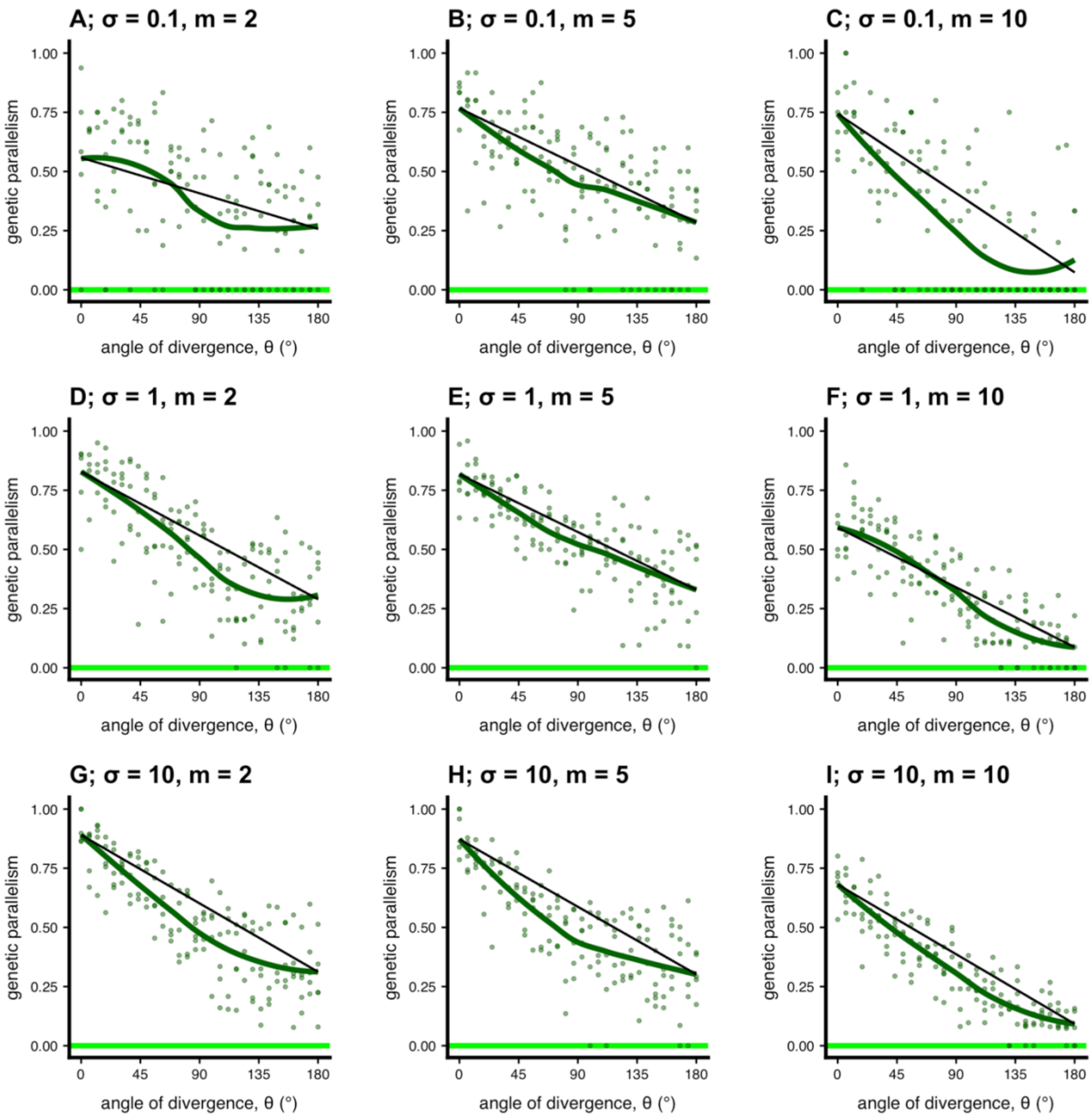

Figure S3. Genetic parallelism across the continuum of parallel to divergent natural selection (N = 5000). This figure presents simulations similar to Fig. 2A in the main text but with varying parameter values (selection [σ] and dimensionality [m]). We ran these particular simulations for T = 1000 generations. All other parameters as in main text. These simulations are computationally intensive and were therefore not run for as many replicates as those plotted in Fig. S2 or S3.

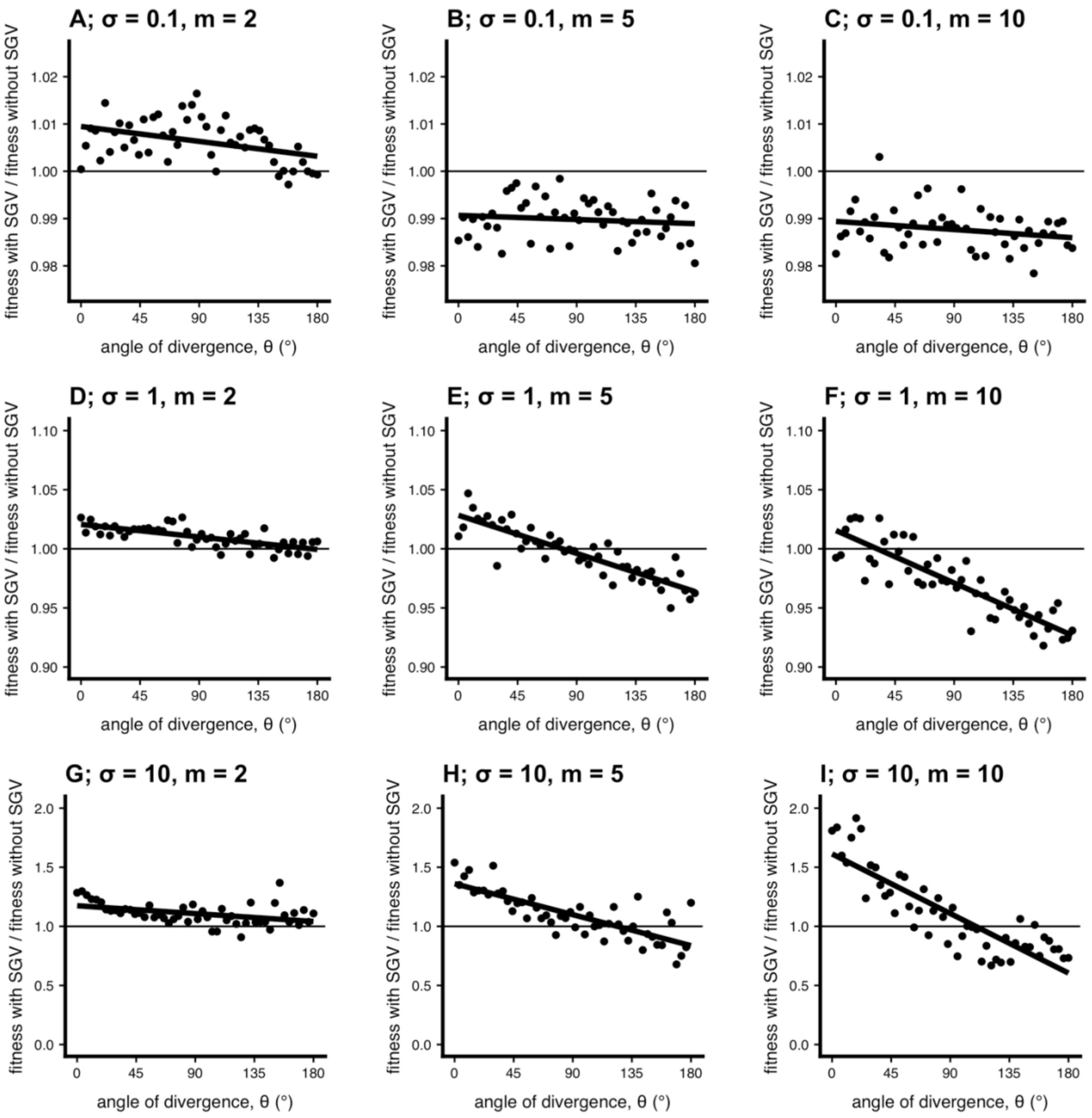

Figure S4. Effect of standing genetic variation on hybrid fitness across the continuum of parallel to divergent natural selection (N = 100). This figure presents simulations similar to Fig. 4B in the main text but with varying parameter values (selection [σ] and dimensionality [m]). We ran these particular simulations for T = 5000 generations. All other parameters as in main text. Note different y-axis scales across rows.

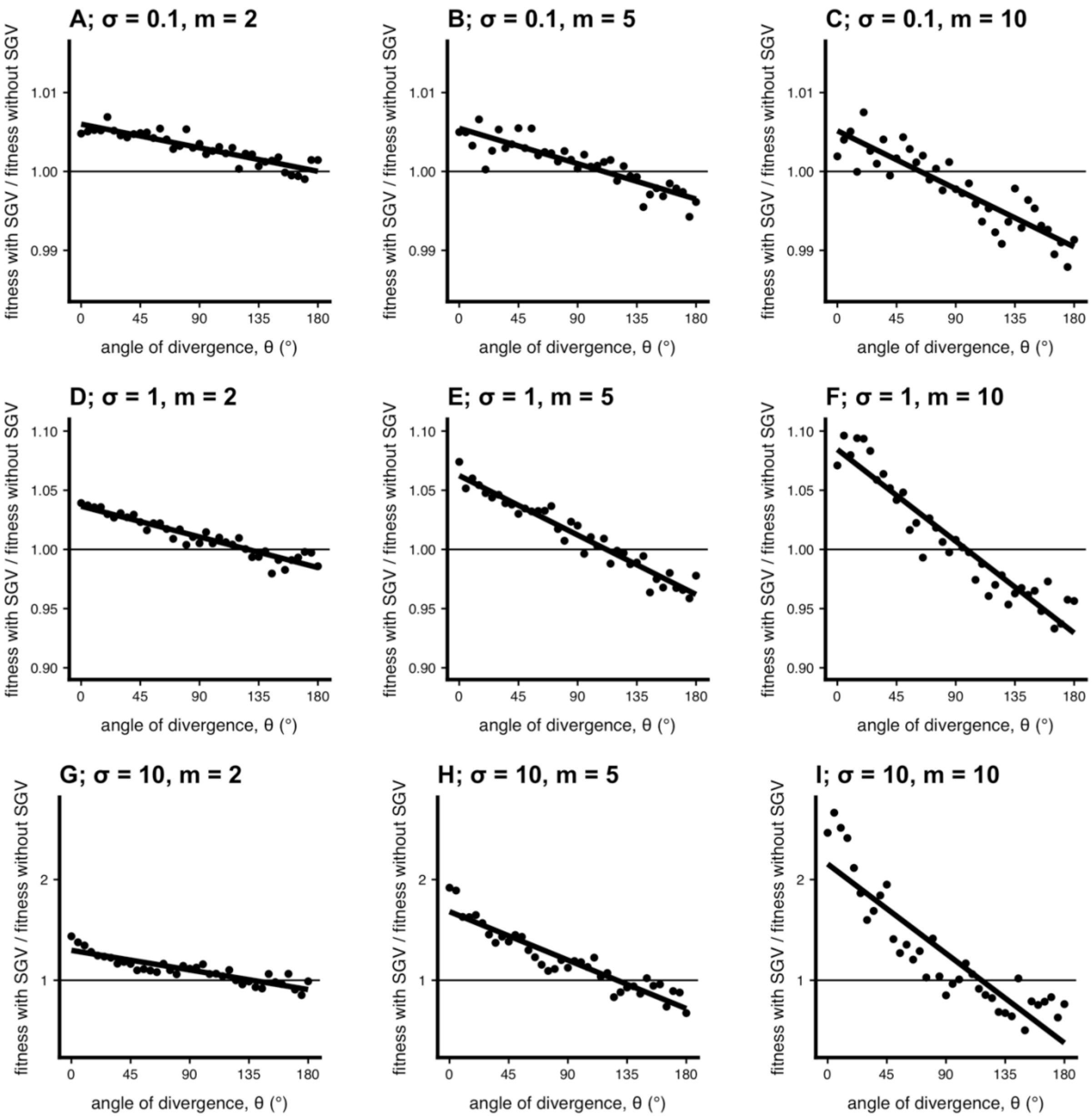

Figure S5. Effect of standing genetic variation on hybrid fitness across the continuum of parallel to divergent natural selection (N = 1000). This figure presents simulations similar to Fig. 4B in the main text but with varying parameter values (selection [σ] and dimensionality [m]). We ran these particular simulations for T = 2000 generations. All other parameters as in main text. Note different y-axis scales across rows.

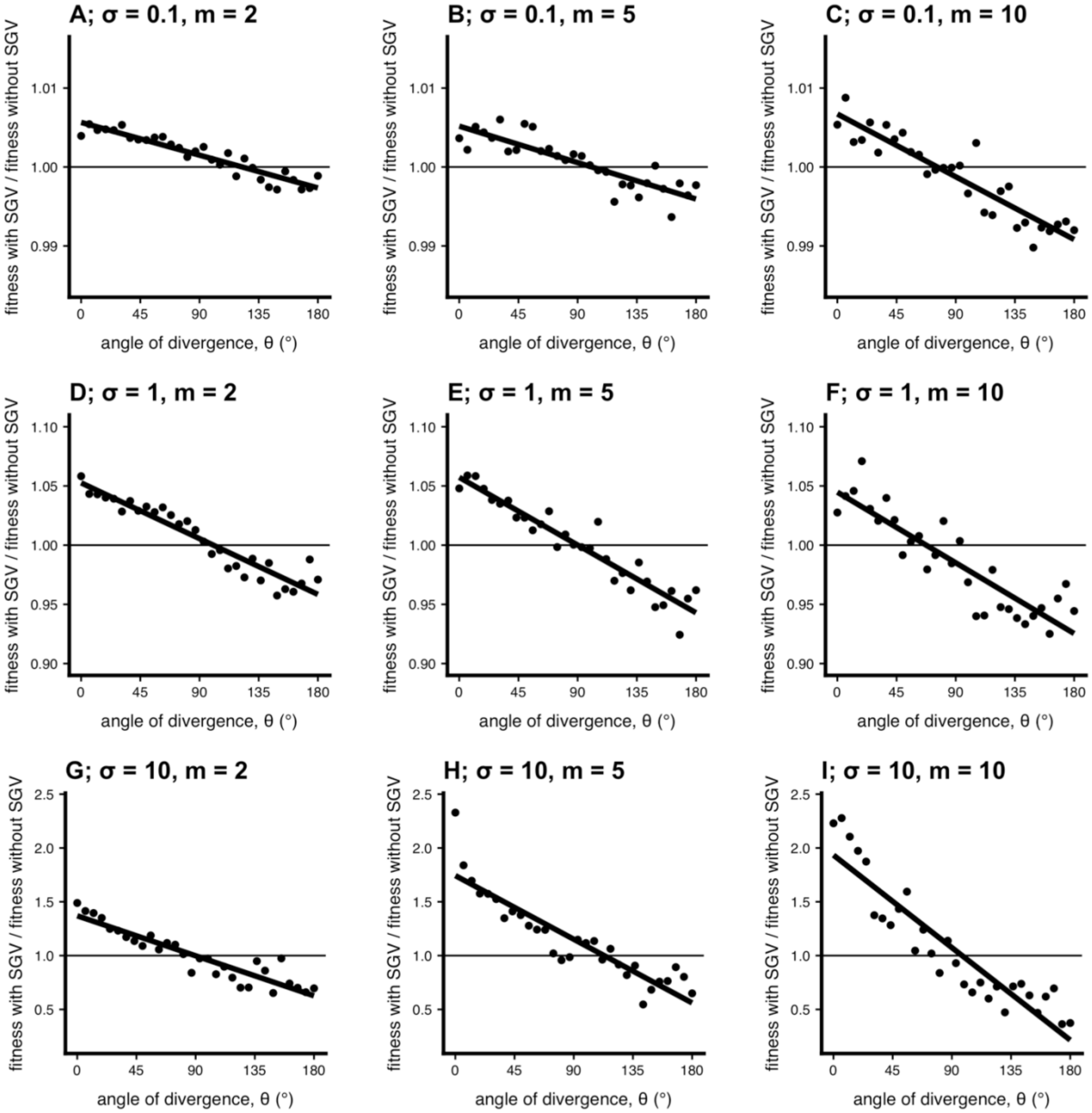

Figure S6. Effect of standing genetic variation on hybrid fitness across the continuum of parallel to divergent natural selection (*N* = 5000). This figure presents simulations similar to Fig. 4B in the main text but with varying parameter values (selection [σ] and dimensionality [m]). We ran these particular simulations for T = 1000 generations. All other parameters as in main text. Note different y-axis scales across rows.

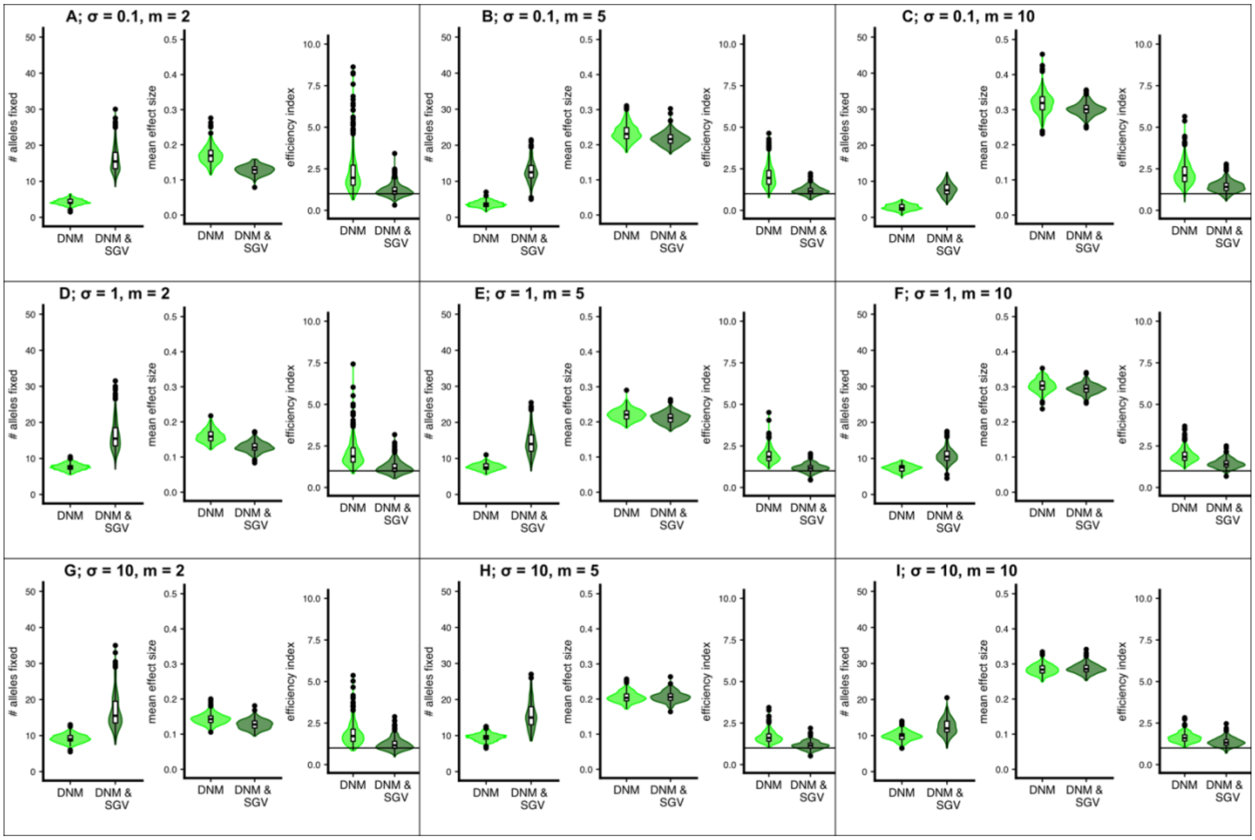

Figure S7. Properties of fixed mutations under a variety of parameter combinations (N = 1000). This figure presents simulations similar to Fig. 6 in the main text but with varying parameter values (selection [σ] and dimensionality [m]). See main text and panel description of Fig. 6 for more detail. Patterns were similar for other population sizes.

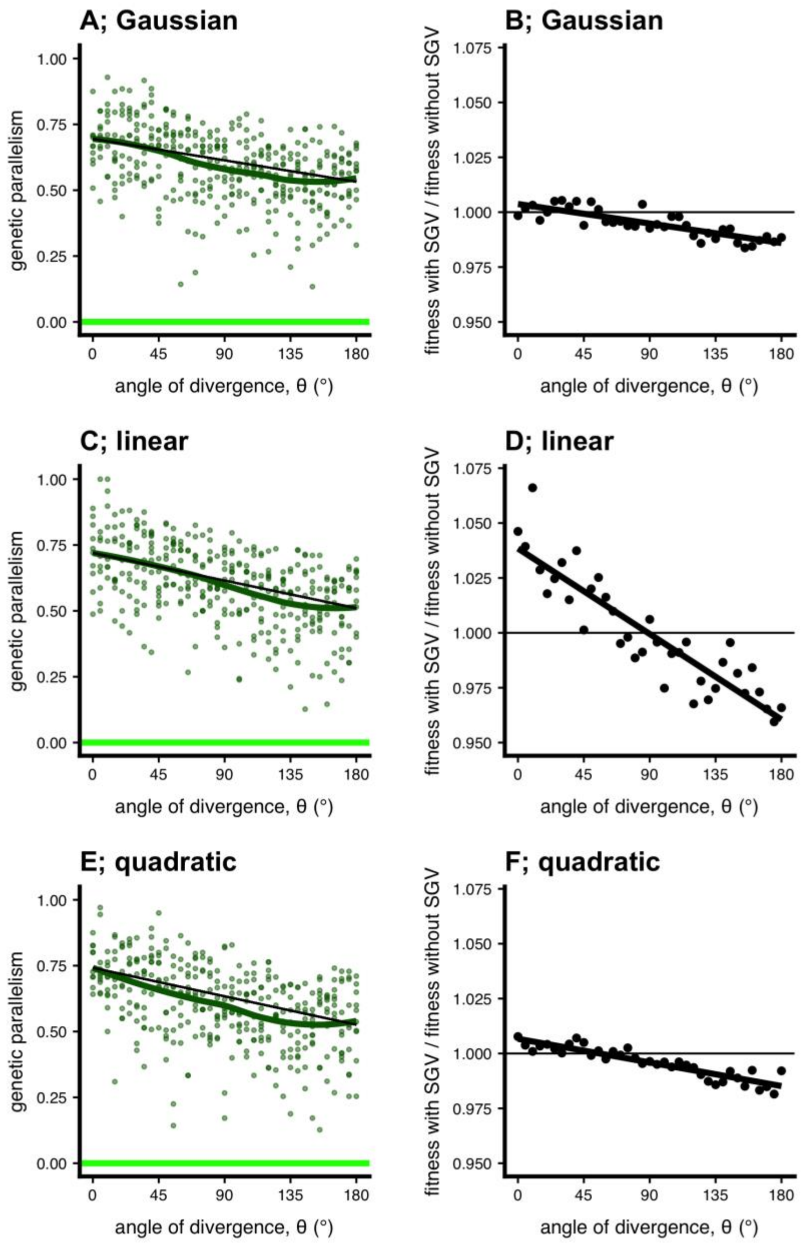

Figure S8. Simulations under various fitness functions. Here we plot simulations across environments for (A & B) Gaussian ($W = \exp(-\sigma \, ||\mathbf{z} - \mathbf{o}||^2/2)$; equation 1), (C & D) linear ($W = 1 - \sigma \, ||\mathbf{z} - \mathbf{o}||$), and (E & F) quadratic ($W = 1 - \sigma \, ||\mathbf{z} - \mathbf{o}||^2/2$) fitness functions. We show results for both genetic parallelism and the effect of standing variation on hybrid fitness. We ran these simulations with a nearer optimum and weaker selection ($d = 0.5$, $\sigma = 0.5$, $N = 1000$, $m = 5$) because populations otherwise became extinct with linear/quadratic fitness functions. Under these conditions, the non-linear decrease in parallelism is less substantial for all parameter values. Nevertheless, the patterns are qualitatively similar among the three sets of simulations (note differences in *y*-axis scales).

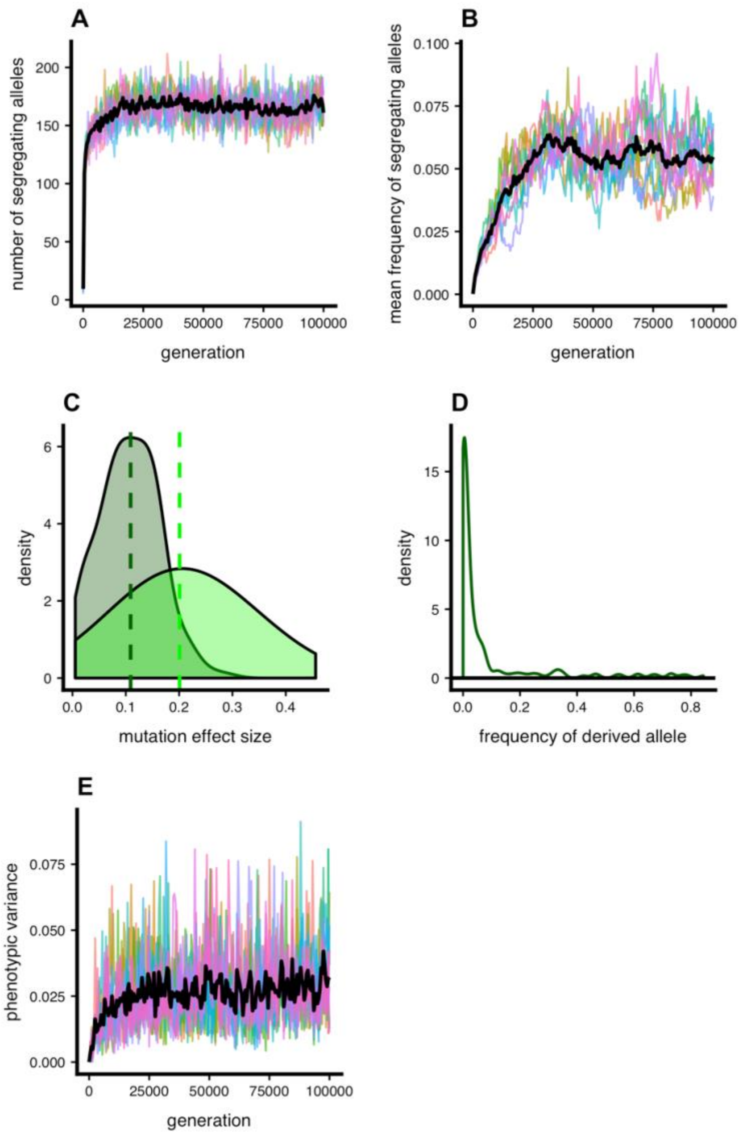

Figure S9. Mutation-selection balance and mutation effect sizes in ancestral populations. In panel (A) we are showing the number of segregating sites in each of 10 ancestral populations and (B) the mean frequency of the derived alleles at each of these sites in the ancestral populations. The black line is plotted through the mean of all populations at each generation, and all ten burn-ins used to generate our main text results are shown. Panel (C) illustrates the distribution of mutation effect sizes—the Euclidean distance of a mutational vector in phenotypic space—at the end of a single representative burn-in simulation (dark green), as compared to the distribution of mutations that arise *de novo* (light green). The vertical lines represent the median mutation effect size for each group. Panel (D) represents the site-frequency spectrum for segregating sites (excluding sites that have fixed). And panel (E) shows the phenotypic variance in the ancestral population over time. (σ = 0.01; m = 5 for all simulations shown; for rest of parameters see Table 1).

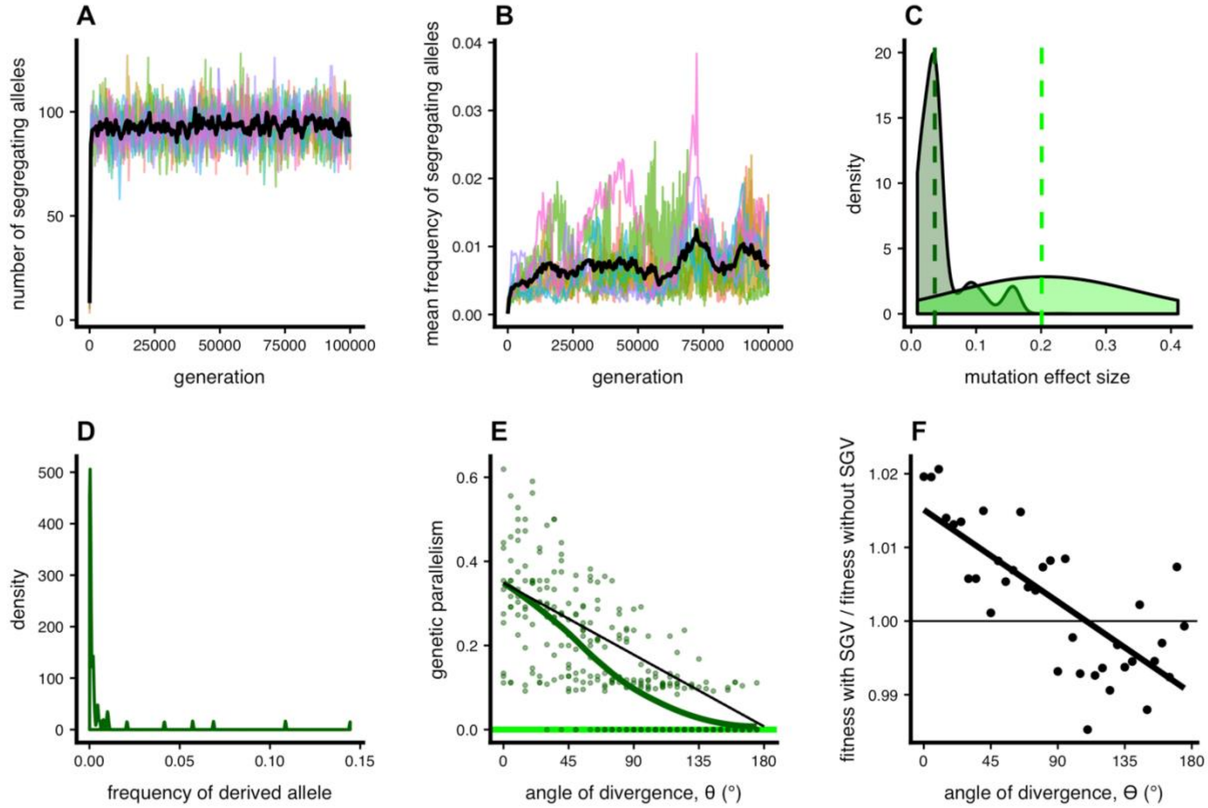

Figure S10. Mutation-selection balance and mutation effect sizes in ancestral populations under stronger selection (σ_{anc} = 1). These parameter values imply $\mu << \alpha^2\ \sigma$, as in the House-of-Cards regime (Turelli 1984, 1985) from a Gaussian regime under an alternative set of parameters. (A) The number of segregating sites in each of 10 ancestral populations and (B) the mean frequency of derived alleles at each of these sites in the ancestral populations. The black line is plotted through the mean of all populations at each generation, and all ten burn-ins used to generate the results ([e] and [f]) are shown. Panel (C) illustrates the distribution of mutation effect sizes—the absolute value of a mutation's effect on the phenotype—at the end of a single burn-in simulation, as compared to the distribution of mutations that arise *de novo*. The vertical lines represent the median mutation effect size for each group. Panel (D) represents the site-frequency spectrum histogram for segregating sites. (Compare these to Fig. S9). Panels (E) and (F) are as in Fig. 2A and 4B in the main text. For unspecified parameters see Table 1 in the main text. This parameter combination t

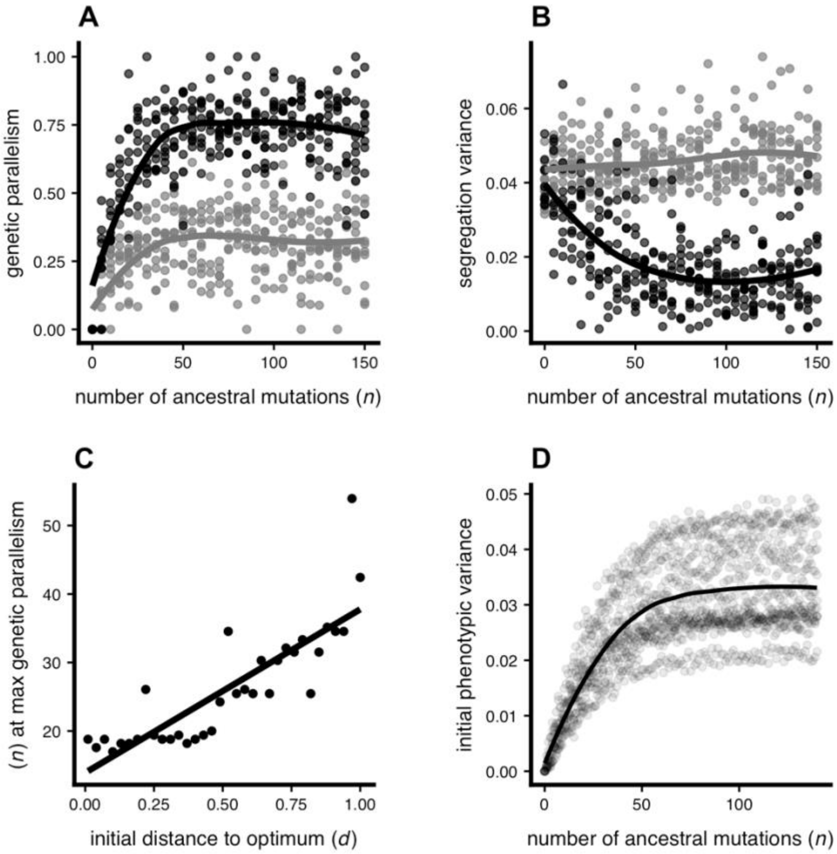

Figure S11. The effects of standing genetic variation on genetic parallelism and phenotypic segregation variance in hybrids under parallel and divergent natural selection. We show (A) genetic parallelism (main text equation 2) and (B) net segregation variance for populations founded with varying quantities of ancestral standing variation (*n*: number of ancestral mutations). Populations were subject to either parallel (θ = 0°; black) and divergent (θ = 180°; grey) selection, with d=1, and there were 10 replicate simulations per parameter combination. Genetic parallelism values of 0 indicate no parallelism and values of 1 indicate complete parallelism (main text Eq. 2). The curves are loess fits. Panel (C) shows that the quantity of ancestral standing variation that maximizes genetic parallelism under parallel selection (θ = 0°) increases when populations adapt to more distant optima. A value of d = 1 is 10 mutational SDs. The line is a linear regression. Panel (D) shows the relationship between the genetic (phenotypic) variation in a parental population as a function of *n*.

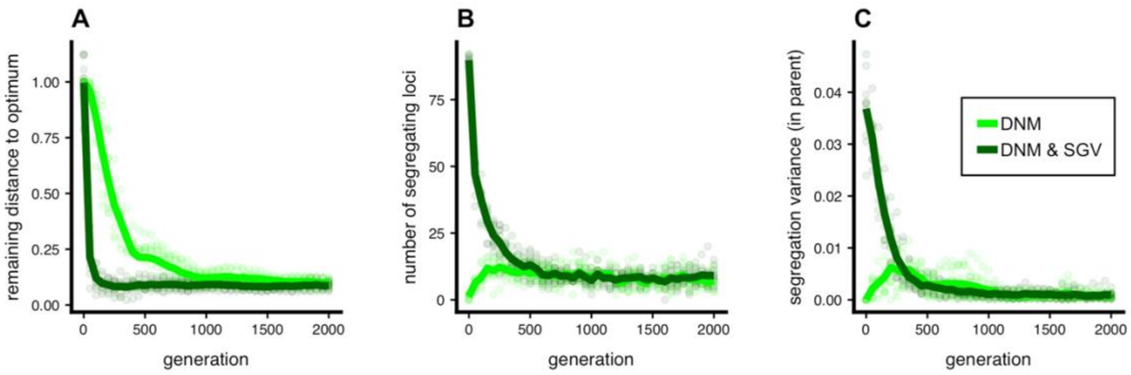

Figure S12. Effect of standing variation on the pace of adaptation and attainment of mutation-selection-drift balance. (A) Populations that adapt with standing variation in addition to new mutation (DNM & SGV; n = 100 segregating alleles; dark green) reach the phenotypic optimum more quickly than populations that adapt from new mutation only (DNM; n = 0 segregating alleles; light green). (B) Although populations equipped with standing variation adapt more quickly than populations adapting from new mutation only, they both reach mutation-selection-drift balance by generation 2000. (C) The phenotypic (genotypic) variance in parental populations, calculated as it is in hybrids (see main text), is stable and near zero by the end of each simulation. The initial distance to the optima, d, is 1 for all simulations. We plot 10 replicate simulations, and lines connect the mean values at each sampled generation. For unspecified parameters see Table 1 in the main text.

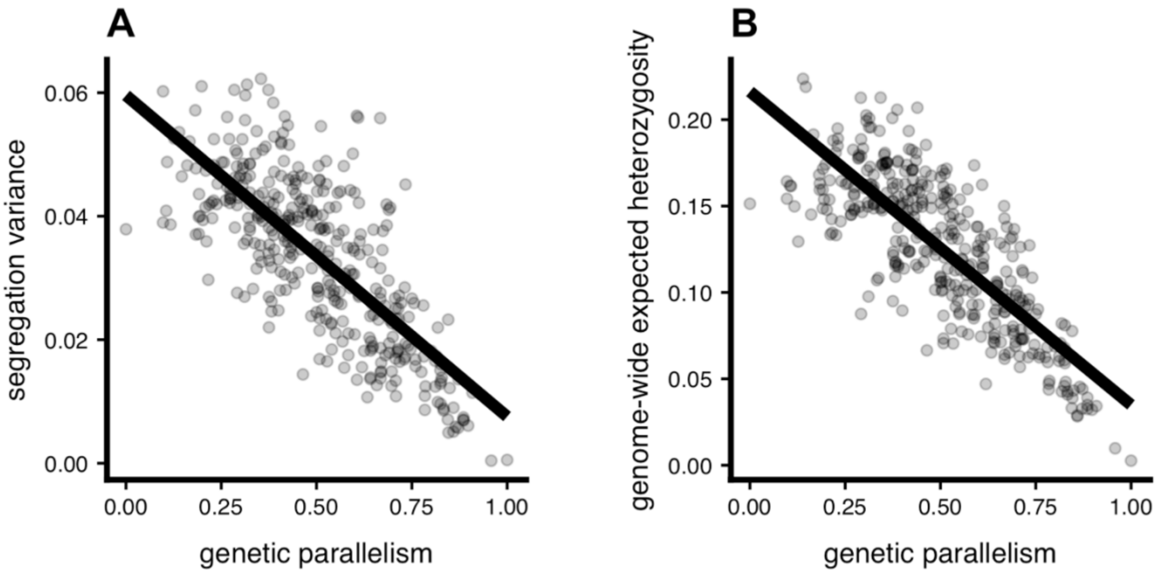

Figure S13. Relationship between genetic parallelism and (A) segregation variance and (B) expected heterozygosity. Our metric of genetic parallelism (main text equation 2) is on the *x*-axis. This is the data plotted in Fig. 2A & 2C of the main text. We show the correlation between genetic parallelism and (A) segregation variance ($r^2 = 0.56$) and (B) genome-wide expected heterozygosity ($2p[1-p]$, averaged across all loci ($r^2 = 0.63$). Patterns were similar for F_{ST} (Hudson et al. 1992) and net π (Nei and Li 1979) (not shown).

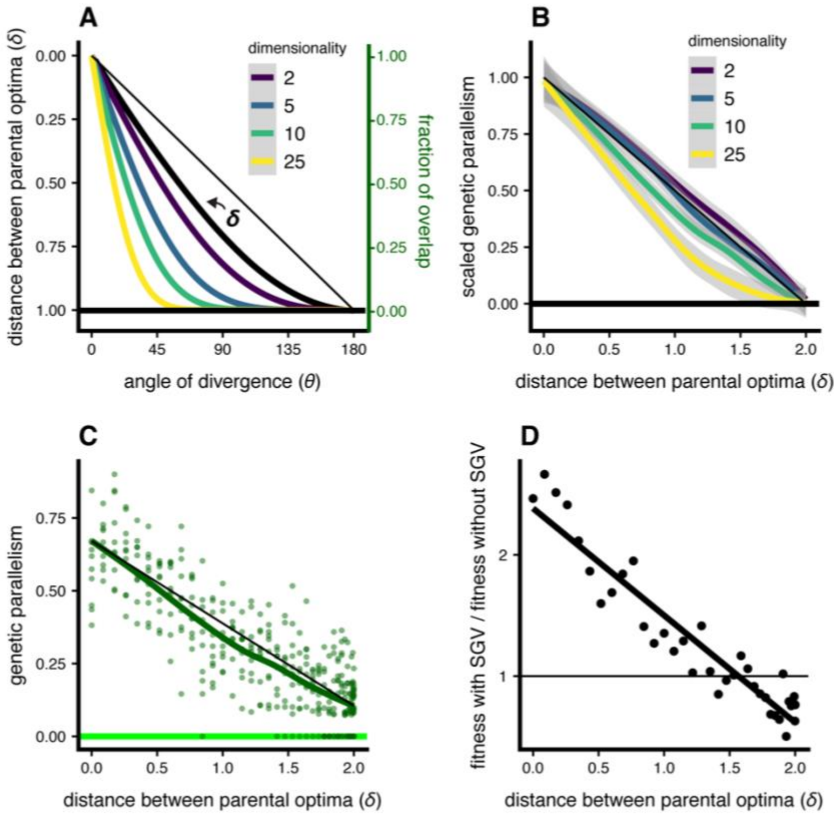

Figure S14. Alternative presentation of simulation results across environments: distance between optima (δ). Panel (A) plots the relationship between the angle of divergence, θ, and the Euclidean distance between parental optima, δ (thick black line; note reversal of left y-axis; scaled between 0 and 1 by dividing by 2d). We also plot the fraction of non-overlap (right [green] y-axis) as in the main text Fig. 3A for four different dimensionalities (m; coloured lines). Panel (B) shows observed (scaled) genetic parallelism vs. δ for the same dimensionalities as plotted in (A). For a given value of θ, δ is invariant with dimensionality (i.e., the distance between optima does not change as dimensionality increases). Accordingly, the nonlinearity emerges even when considering δ, but only appreciably when considering higher dimensions (m > 5). In both panels, the thin and straight black line connects the fit at 0° with 180° for visual reference. In panels (C) and (D) we show the raw data for genetic parallelism and relative hybrid fitness in simulations conducted for simulations conducted 10 dimensions (m = 10, σ = 1, N = 1000).

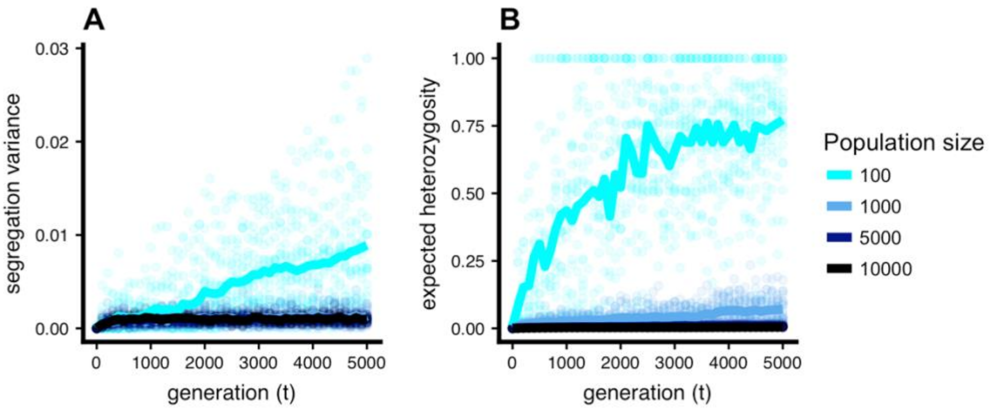

Figure S15. The effect of population size on the rate of divergence between populations due to drift. We show populations held at a common optimum with no standing variation (i.e. $d = 0$, $n = 0$) and plot (A) segregation variance and (B) expected heterozygosity in hybrids over time for 5,000 generations. The evolution of segregation variance is proportional to the rate of evolution of reproductive isolation under parallel natural selection. Greater drift in smaller populations leads to greater segregation variance and heterozygosity. The lines are drawn as the average of 10 replicate simulations ($m = 5$, $\sigma = 1$).

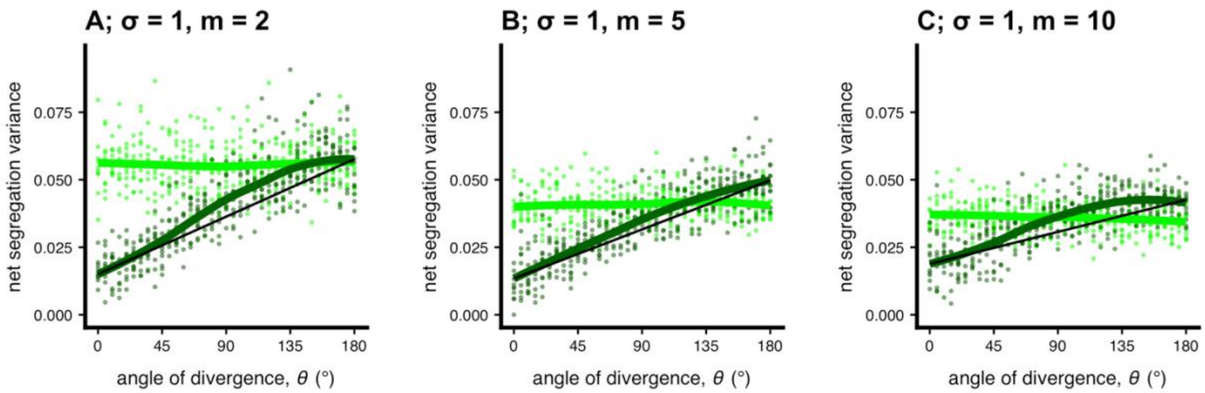

Figure S16. Effect of dimensionality on net segregation variance. These plots are similar to Fig. 2C in the main text except we show results for three different dimensionalities. Under divergent natural selection, simulations where populations adapted from standing variation (dark green) had higher segregation variance—relative to simulations where populations adapted only from *de novo* mutation (light green)—in higher dimensions. Note the overall trend of a decrease in net segregation variance as dimensionality increases.

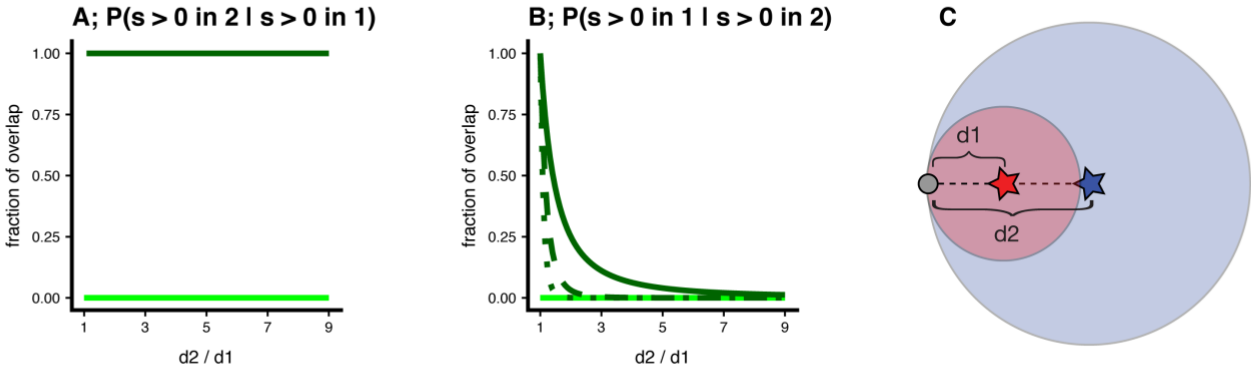

Figure S17. Fraction of overlap of beneficial mutations with parallel selection ($\theta = 0°$) but unequal distance ($d_1 \neq d_2$). The main text explores how the fraction of overlap changes with theta while holding $d_1 = d_2 = d$ constant. Here we explore how the fraction of overlap changes with the ratio d_2 / d1 when holding $\theta = 0°$ constant. Unlike the metric presented in the main text this metric is asymmetrical because one population is completely contained within the other. Panel (A) plots the fraction of overlap for population 1 (the fraction of alleles that are beneficial in population 1 are also beneficial in population 2) as a function of d_2 / d_1. With $d_1 < d_2$ the value is 1 for any ratio d_2 / d_1 because population 1's hypersphere is contained within population 2's. Panel (B) plots the fraction of alleles that are beneficial in population 2 that are also beneficial in population 1. This latter result mirrors what is seen in the main text Fig. 3A: as the locations of the optima depart from one another the fraction of overlap rapidly approaches zero and does so most rapidly at the onset of departure. Panel (C) shows a cartoon example of a case in 2-dimensions where $d_2 = 2d_1$.

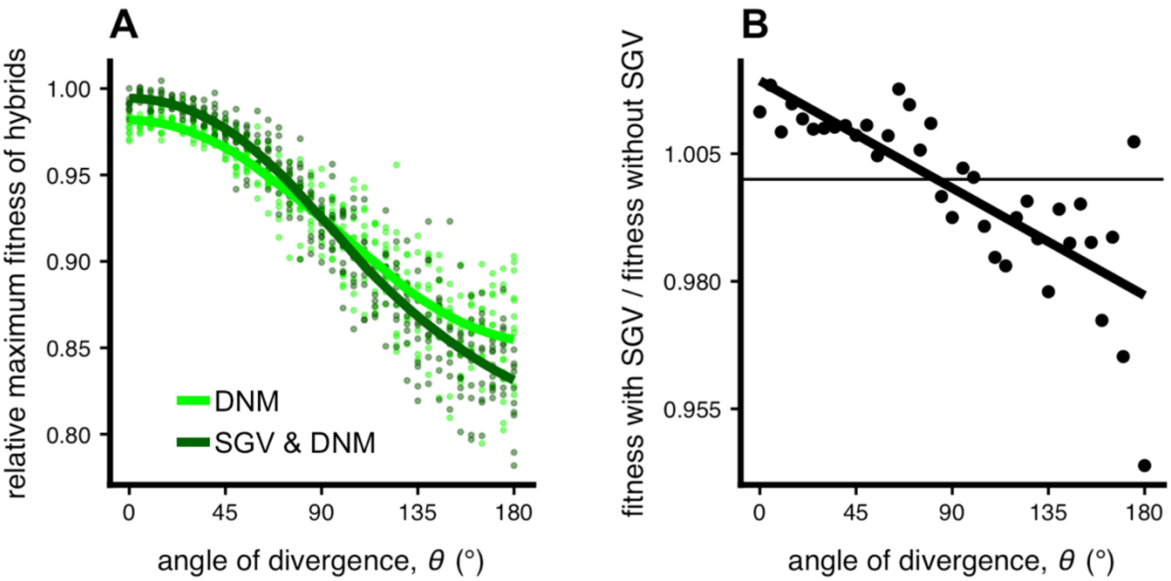

Figure S18. The effect of standing genetic variation (SGV) on relative maximum hybrid fitness across environments. Data are from simulations plotted in the main text, but instead of mean fitness of all hybrids we depict the mean fitness of the top 5 % of hybrids relative to the mean fitness of parents. We plot both the (A) raw values of relative maximum fitness and (B) the effect of standing variation on maximum hybrid fitness (dark green divided by light green).

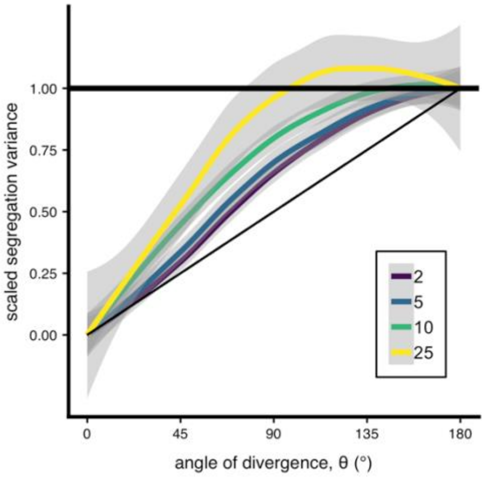

Figure S19. The relationship between segregation variance and θ for different dimensionalities. We plot the loess fits of proof-of-concept simulation results with 95 % confidence intervals conducted in four different dimensionalities (colours), each scaled between 0 (at 0°) and 1 (at 180°). Simulations were conducted with strong natural selection (σ = 10) to minimize the effect of drift.
